# Dynamic PET-Based Networks: A Critical Review of Reactive Strategies, Processing Windows, and Circular Applications

**DOI:** 10.1021/acsomega.6c04153

**Published:** 2026-10-29

**Authors:** Amanda Araújo, José Barreto, Lorena Dantas, Euller Sousa, Carlos Luna, Danilo Siqueira, Edcleide Araújo, Marta Fernández-García, Renate Wellen

**Affiliations:** † Department of Chemistry, Federal University of Paraíba, João Pessoa, Paraíba 58051-900, Brazil; ‡ Department of Design, Federal University of Campina Grande, Campina Grande, Paraíba 58429-900, Brazil; § Mackenzie Graphene and Nanomaterials Research Center, Mackenzie Presbyterian University, São Paulo, São Paulo 01302-907, Brazil; ∥ Instituto de Ciencia y Tecnología de Polímeros, Madrid 28006, Spain; ⊥ Materials Engineering, Federal University of Paraíba, João Pessoa, Paraíba 58051-900, Brazil

## Abstract

Poly­(ethylene terephthalate) (PET) is an industrial commodity, with global production reaching ∼82 million metric tons in 2024 and a projected market value of USD 40.66 billion by 2025. Despite its ubiquity, PET circularity is hindered by thermomechanical degradation during recycling. This review addresses a significant gap by integrating chemical design, semicrystalline morphology, and industrial scalability. A comparison of recent developments reveals that while imine, disulfide, and boronic chemistries are widely researched, they fail in PET due to moisture sensitivity and thermal instability. Ester-based transesterification is established as the only mechanism intrinsically compatible with the PET backbone. A key highlight is that PET vitrimer behavior is morphology-limited rather than chemistry-limited. With crystallinity levels between 25% and 45%, exchange reactions are confined to the amorphous phase, resulting in heterogeneous stress relaxation and high activation energies. Furthermore, stress relaxation alone is an insufficient validation metric; molecular weight retention must be monitored to ensure flow originates from associative exchange rather than degradation. Regarding scalability, reactive extrusion (RE) is the primary industrial platform. Success depends on moisture management, defined as a first-order design variable to prevent hydrolysis within narrow processing windows. By balancing exchange kinetics against degradation, this work provides actionable principles for the scalable upcycling of PET into high-performance materials for a circular economy.

## Graphical abstract

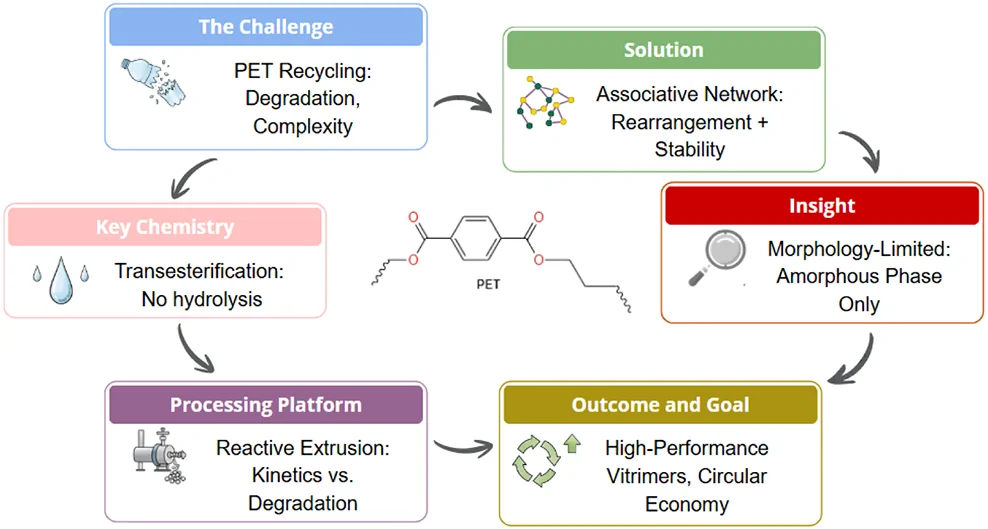

## 1 Introduction

Poly­(ethylene terephthalate) (PET) is one of the most widely produced and consumed thermoplastics worldwide. According to consolidated industrial and market data, global PET resin production reached approximately 82 million metric tons in 2024,^1^ positioning PET as one of the most widely manufactured polymers globally. These attributes underpin its extensive use in packaging, textile fibers, automotive components, and engineering applications, and simultaneously highlight the magnitude of PET-derived waste streams and their associated environmental impact.^2–4^

In economic terms, PET also represents a major industrial commodity. The global polyethylene terephthalate market is projected to reach approximately USD 40.66 billion by 2025,^5^ reflecting its central role in packaging, textiles, and consumer-goods’ supply chains. This combination of large production volumes and high economic value underscores the importance of developing robust strategies for PET reuse, recycling, and upcycling within a circular-economy framework.^6–9^

Conventional mechanical recycling remains the dominant route for PET waste management. However, repeated melt-processing cycles are well known to induce thermomechanical degradation, leading to chain scission, molecular weight reduction, and progressive deterioration of mechanical, rheological, and optical properties.^10–12^ As a result, mechanically recycled PET often exhibits inferior performance compared with virgin material, limiting its reuse in high-value applications. Chemical recycling approaches, including glycolysis, methanolysis, and hydrolysis, can recover monomers or oligomeric intermediates but typically require elevated temperatures, high pressures, and complex purification steps, which constrain their economic and industrial viability. These intrinsic limitations have driven increasing interest in alternative recycling and upcycling concepts capable of preserving polymer value across multiple processing cycles.^13–16^

Within this context, vitrimers have emerged as a distinct class of polymer networks that bridge the gap between thermoplastics and thermosets. Since their introduction by Leibler and coworkers in 2011,^17^ vitrimers have been defined by permanent polymer networks capable of undergoing topological rearrangement through associative dynamic covalent bond exchange reactions, enabling stress relaxation and macroscopic flow at elevated temperatures without loss of cross-link density.^18–20^ This unique combination allows vitrimers to be reshaped, welded, repaired, and recycled while retaining the dimensional stability and solvent resistance characteristic of thermoset materials.^21–23^

Over the past decade, research activity on vitrimer materials has expanded substantially, encompassing a wide range of dynamic covalent chemistries, including transesterification, transamination, imine exchange, disulfide metathesis, boronic ester exchange, and metal–ligand coordination.^24–31^ Bibliometric analyses based on established scientific databases consistently report a marked increase in peer-reviewed publications on vitrimers and dynamic covalent polymer networks since the mid-2010s, reflecting the consolidation of this field as a major research direction in contemporary polymer science.^32–34^

Among the various vitrimer chemistries, ester-based exchange reactions are particularly relevant for PET and related polyester systems, as ester functionalities are intrinsic to the polymer backbone. This intrinsic chemical compatibility enables vitrimer behavior to be introduced without extensive modification of the base polymer structure, making polyester vitrimers especially attractive for PET upcycling and reprocessing strategies.^35–38^ At the same time, PET presents specific challenges due to its semicrystalline nature, as crystalline domains restrict chain mobility and strongly influence stress relaxation, exchange kinetics, and macroscopic flow behavior.^39–41^

The vitrimer response of PET-based systems is therefore governed by a complex interplay among network architecture, catalyst type and concentration, processing temperature, shear history, and degree of crystallinity. Insufficient control over these parameters can result in heterogeneous network structures, incomplete exchange reactions, excessive chain scission, or degradation during reprocessing, ultimately compromising mechanical performance and recyclability.^42–48^

Reactive extrusion (RE) has been widely reported as a viable processing route for the preparation and reprocessing of PET-based vitrimer systems, owing to its continuous operation, short residence times, and compatibility with existing thermoplastic processing infrastructure.^49–52^ However, the simultaneous application of high temperatures and shear stresses inherent to RE processing can promote both dynamic bond exchange and degradation. Establishing robust processing windows that balance exchange kinetics, network stability, and resistance to degradation, therefore, remains a central challenge for the scalable implementation of PET vitrimers.^13,52–54^

Several review articles have addressed vitrimers from a general perspective, focusing on dynamic covalent chemistries, exchange mechanisms, and applications such as self-healing materials, coatings, and adhesives.^55–58^ Other reviews have examined polyester recycling and upcycling strategies without explicitly addressing vitrimeric behavior or the processing–structure–property relationships specific to dynamic networks.^59–61^ To date, however, a critical, integrated review dedicated specifically to PET-based vitrimer systems, simultaneously considering chemical design, processing routes, structure–property relationships, and scalability constraints, remains lacking.

The present review addresses this gap by providing a critical and systematic analysis of PET-based vitrimer systems and related dynamic polyester networks. Emphasis is placed on ester-based associative exchange mechanisms, their interaction with PET’s semicrystalline structure, and their response to processing conditions, particularly during reactive extrusion. By consolidating and critically analyzing dispersed data from the literature data, this review aims to establish robust design principles for PET vitrimer systems and support the development of scalable, high-performance materials aligned with circular polymer economy objectives.

## 2 Systematic Literature Review Methodology

### 2.1 Review Design and Scope

This review was conducted following the principles of a systematic literature review (SLR),^62,63^ in accordance with the PRISMA 2020 (Preferred Reporting Items for Systematic Reviews and Meta-Analyses) guidelines. The objective was to identify, critically analyze, and synthesize peer-reviewed literature related to PET-based vitrimer systems and dynamic polyester networks, with particular emphasis on ester-based associative exchange mechanisms, processing routes, and structure–property–processing relationships relevant to recyclability and scalability.

The scope of the review was intentionally defined to focus on PET and closely related polyester systems, in which vitrimer behavior was experimentally demonstrated through dynamic covalent bond exchange. The conceptual boundaries of the review, as well as the predefined inclusion and exclusion criteria applied during the selection process, are summarized in Table 1, ensuring a transparent, consistent, and reproducible screening strategy.

**Table 1:** Scope Definition and Eligibility Criteria Adopted in the Systematic Literature Review

Category	Definition applied in this review
Polymer systems	Poly(ethylene terephthalate) (PET)-based systems and closely related polyester networks (e.g., poly(butylene terephthalate) (PBT), poly(butylene succinate) (PBS), poly(lactic acid) (PLA)), when used as comparative or supporting frameworks directly relevant to PET.
Network type	Vitrimers and dynamic covalent polymer networks with experimentally demonstrated associative bond exchange behavior.
Dynamic covalent chemistry	Ester-based exchange reactions (primary focus); imine exchange, disulfide metathesis, boronic ester exchange, and metal–ligand coordination when applied to polyester backbones or discussed in direct comparison with PET systems.
Catalyst systems	Organometallic, metal salt, organic, or coordination-based catalysts explicitly reported to promote dynamic bond exchange.
Processing routes	Batch processing, melt blending, compression molding, and reactive extrusion under laboratory or industrially relevant conditions.
Material characterization	Thermal, mechanical, rheological, stress relaxation, creep, reprocessability, recyclability, and network stability analyses.
Evidence requirements	Explicit experimental validation of vitrimer behavior (e.g., stress relaxation, topology rearrangement, reshaping or reprocessing without loss of cross-link density).
Publication type	Peer-reviewed journal articles.
Language	English.
Excluded materials	Nonpolyester vitrimer systems without direct relevance to PET; purely theoretical or modeling studies without experimental validation.
Excluded document types	Patents, conference proceedings, review articles (except for contextual discussion).

### 2.2 Data Sources and Search Strategy

The literature search was performed using Google Scholar and ScienceDirect, which were selected for their comprehensive coverage of high-impact, peer-reviewed journals in polymer science, materials chemistry, and chemical engineering. Searches were conducted using combinations of keywords related to PET, polyesters, and vitrimer chemistry that were applied to titles, abstracts, and author keywords.

The primary search terms included combinations of:

- *“poly­(ethylene terephthalate)” OR “PET”*
- “vitrimer*”
- “dynamic covalent network*”
- *“transesterification”*
- *“associative exchange”*
- “polyester vitrimer*”
- *“reactive extrusion”*

Boolean operators (AND/OR) were used to refine the queries and ensure the comprehensive retrieval of relevant records. Only peer-reviewed journal articles published in English were considered. Non-peer-reviewed literature, including patents and conference proceedings, was excluded in accordance with the eligibility criteria defined in Table 1.

To minimize the risk of omitting relevant studies, backward and forward citation tracking was performed for key articles identified during the screening process, particularly those that were highly cited or conceptually central to PET-based vitrimer research.

### 2.3 Study Selection and Eligibility Criteria

The study selection was carried out in sequential stages. Initially, all retrieved records were screened based on titles and abstracts to exclude publications that were clearly outside the defined scope. Articles were excluded at this stage if they did not involve PET or polyester-based systems, did not address vitrimers or dynamic covalent network behavior, or focused exclusively on conventional thermoplastics or thermosets.

Subsequently, full-text screening was conducted on the remaining publications. Studies were included only if they met all the eligibility criteria defined in Table 1, namely:

(i) explicit experimental evidence of vitrimer or dynamic covalent network behavior;
(ii) use of PET or polyester backbones, or direct relevance to PET through comparative analysis;
(iii) sufficient methodological detail to support the critical assessment of processing conditions, network structure, and material properties.

### 2.4 Quality Assessment and Data Reliability

The methodological quality and data reliability of the included studies were assessed qualitatively, with an emphasis on transparency and completeness of experimental reporting. Particular attention was given to the description of polymer synthesis and processing routes, the identification of the dynamic exchange mechanisms and catalysts, and the reporting of key properties, such as stress relaxation, mechanical performance, and reprocessability.

Rather than excluding studies based on rigid scoring systems, this Review adopts a critical synthesis approach, in which methodological limitations, uncertainties, and inconsistencies reported in the literature are explicitly acknowledged and discussed. This approach allows identification of general trends while highlighting recurring challenges in PET-based vitrimer research.

### 2.5 Data Extraction and Synthesis

For each selected study, relevant information was extracted regarding the polymer composition, network architecture, dynamic covalent chemistry, catalyst system, processing route, and reported thermal, mechanical, and rheological properties. The extracted data were organized into comparative tables and thematic categories to facilitate cross-study analysis.

Because of variability in experimental protocols and reporting formats in the literature, a quantitative meta-analysis was not performed. Instead, results were synthesized through qualitative and semiquantitative comparisons, focusing on relationships among chemical design, processing conditions, and macroscopic material behavior.

### 2.6 Reporting Transparency

The overall study selection process is summarized using a PRISMA 2020 flow diagram (Figure 1), which details the number of records identified, screened, excluded, and included at each stage of the review process.

**Figure 1:**
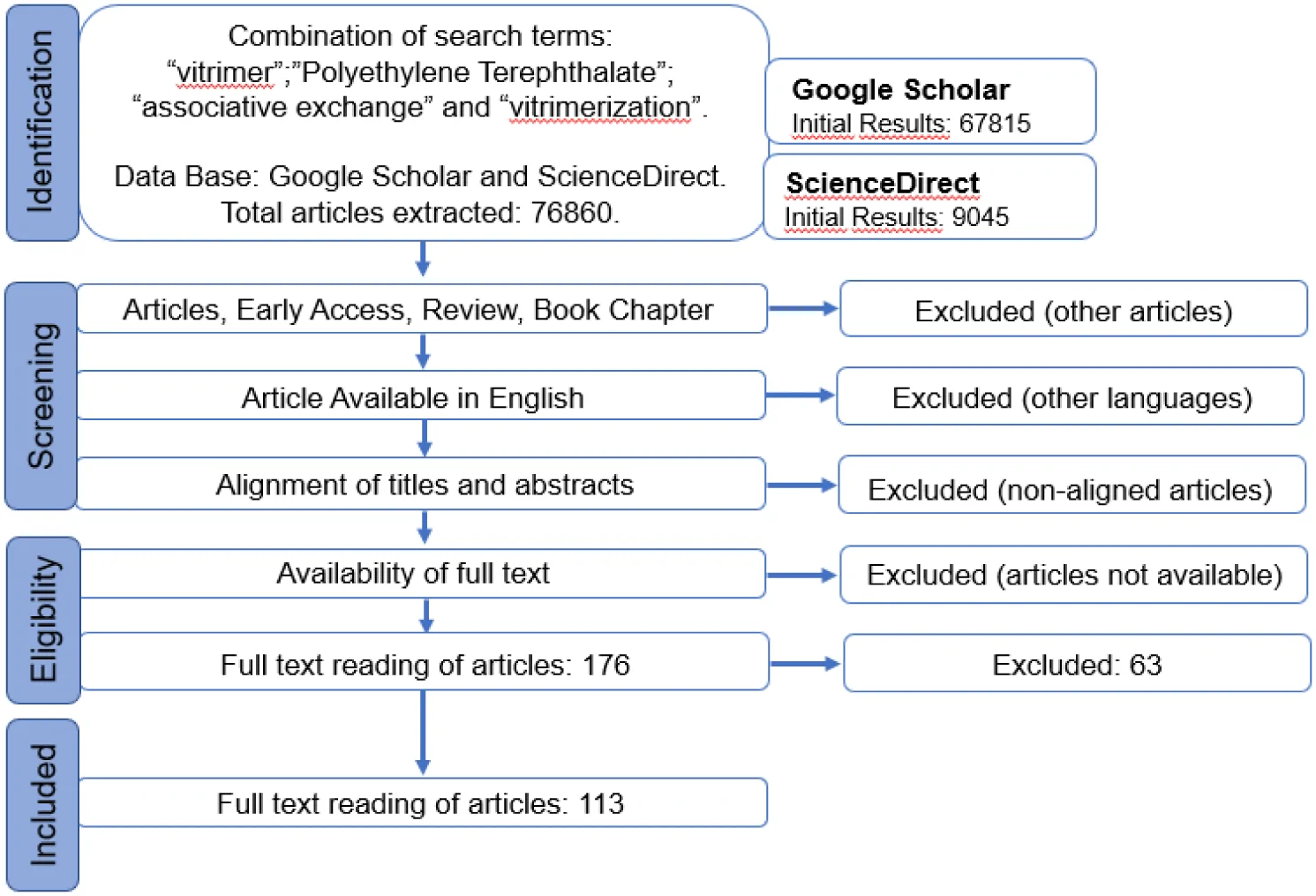
Adapted PRISMA 2020 flow diagram illustrating the systematic literature selection process.

The diagram summarizes the identification of records through database searching and citation tracking, the screening of titles and abstracts, the full-text eligibility assessment, and the final inclusion of studies in the qualitative synthesis. The inclusion and exclusion criteria applied at each stage are defined in Table 1.

This diagram ensures transparency and reproducibility of the literature selection methodology and should be interpreted in conjunction with the eligibility criteria presented in Table 1.

## 3 Ester-Based Exchange Mechanisms in Dynamic PET Based Networks

Covalent adaptable networks (CANs) are a broad category of cross-linked polymers that utilize dynamic covalent bonds to rearrange their topology in response to external stimuli.^21,35^ These systems are classified, based on their exchange pathways, into dissociative CANs and associative CANs (also known as vitrimers).^13,21,64,65^ Dynamic PET-based networks utilize these principles to upcycle waste polyethylene terephthalate into high-performance, recyclable materials. While many dynamic PET-based networks are engineered specifically as vitrimers through associative transesterification or imine exchange, the broader classification of dynamic PET-based networks encompasses both pathways, allowing for the design of either dissociative or associative architectures, depending on the specific dynamic chemistries employed.^6,35,36,51^

Hayashi et al.^66^ studied the transformation of linear polyesters into vitrimers using multifunctional epoxy cross-linkers and base catalysts during reactive processing. These studies focused on the sequential mechanisms of chain decomposition, epoxy ring-opening, and network formation via transesterification. They demonstrated this behavior by identifying that initial chain scission is a critical step to increase the fraction of reactive end-groups, which subsequently enables the formation of branched, dangling points and a robust dynamic network.

### 3.1 Transesterification as a Core Dynamic Exchange Reaction

Ester-based exchange reactions constitute the most chemically intrinsic and technologically relevant dynamic covalent mechanism for the development of PET-based vitrimer systems, since ester linkages are the fundamental structural motif of the PET backbone. Polyester vitrimer behavior is predominantly enabled by associative transesterification reactions, allowing dynamic exchange of ester bonds while maintaining constant cross-link density and network connectivity, thereby preserving dimensional integrity during thermal activation,^17^ as shown in Figure 2.

**Figure 2:**
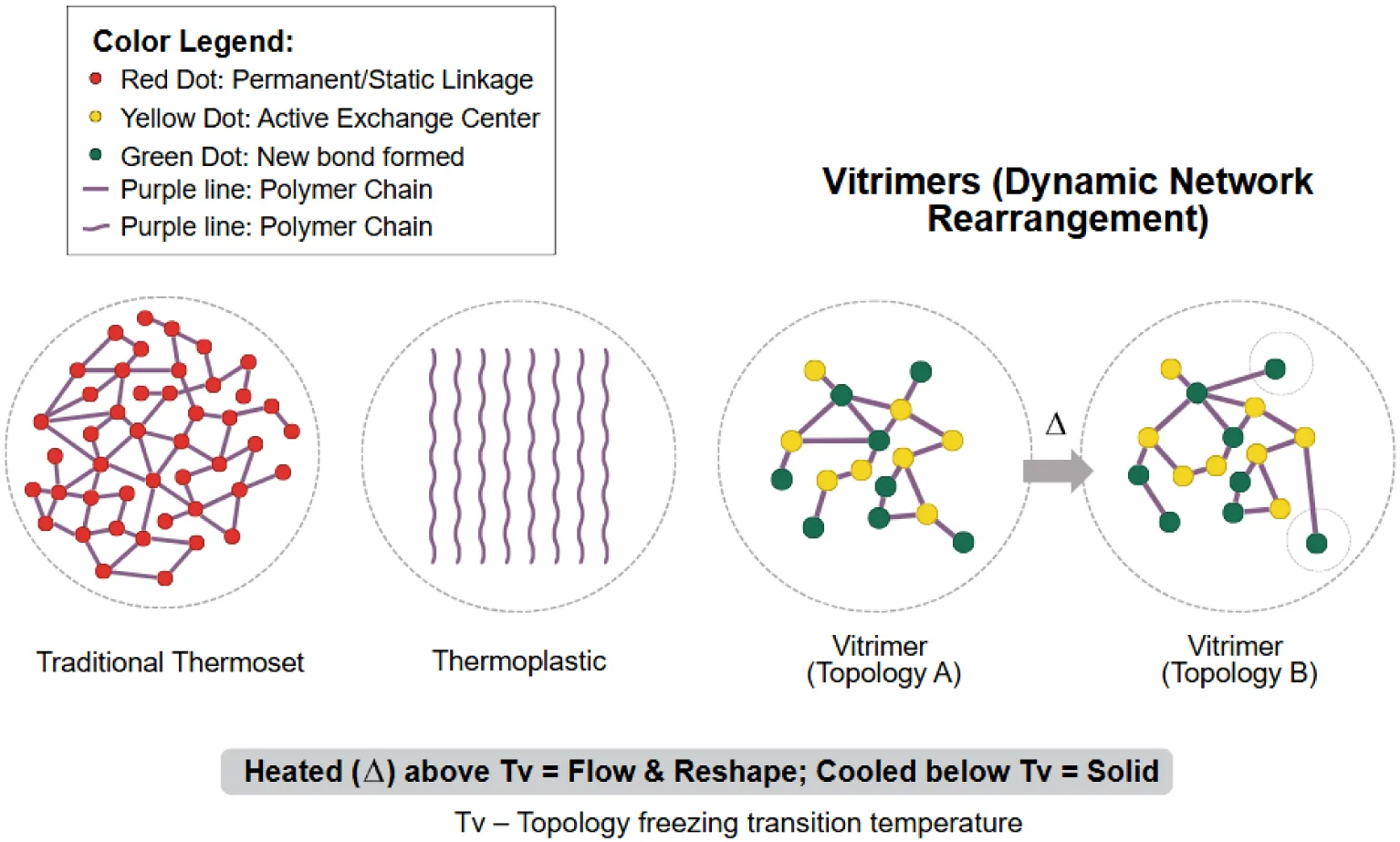
Schematic representation of network topology and dynamic rearrangement in vitrimer systems, compared with conventional polymer classes (thermosets and thermoplastics).

Ester exchange, also known as transesterification, is a dynamic covalent reaction that proceeds through the addition of an oxygen nucleophile to a carbonyl group. Chemically, this process is characterized by its robustness, typically requiring the use of strong acid or base catalysts to facilitate the exchange of the alcohol moiety. Specifically, Brønsted bases such as NaO*t*-Bu (sodium tert-butoxide) and DBU (1,8-diazabicyclo[5.4.0]­undec-7-ene), as well as Lewis acids like Ti­(OBu)_4_, are used to promote equilibration. Schaufelberger et al.^24^ highlight a significant disparity between esters and other dynamic bonds: while thioester exchange can occur under mild conditions in water without catalysis, ester exchange requires harsh conditions and often a high excess of base to achieve practical equilibration rates. This requirement for high catalyst loading can be problematic, as it may lead to the undesired decomposition of system components during prolonged exchange or alter the equilibrium position due to complexation effects.

From a mechanistic standpoint, transesterification in vitrimeric polyesters proceeds via a nucleophilic acyl-substitution mechanism, involving the attack of a hydroxyl group on the carbonyl carbon of an ester moiety, followed by the formation of a tetrahedral intermediate and subsequent exchange of alkoxy groups. The associative nature of this mechanism, where a new bond is formed before the original bond is cleaved, is a defining characteristic of vitrimer systems, fundamentally distinguishing them from dissociative CANs and ensuring the absence of a sol–gel transition upon heating.^67^

Traditional thermosets exhibit permanently cross-linked networks with static covalent bonds, whereas thermoplastics consist of linear or branched polymer chains without permanent cross-links. In vitrimer networks, permanent covalent cross-links are preserved, but associative exchange reactions enable topological rearrangement of the network without loss of cross-link density. Red dots represent permanent/static linkages, yellow dots indicate active exchange centers, and green dots correspond to newly formed bonds after the exchange (see Figure 2).

In PET-based systems, the feasibility of transesterification is enhanced by the presence of intrinsic hydroxyl and carboxyl end groups, generated during polycondensation, and further increased during mechanical recycling as a result of chain scission. These terminal functionalities act as active nucleophilic sites, enabling exchange reactions even without extensive chemical prefunctionalization of the polymer chains.^68^

From a thermodynamic perspective, transesterification reactions in polyesters are typically close to equilibrium, implying that vitrimer behavior is governed primarily by kinetic control rather than thermodynamic driving forces. Consequently, the efficiency of bond exchange depends strongly on temperature, catalyst activity, and local chain mobility. This kinetic regime explains the Arrhenius-type temperature dependence commonly observed for stress relaxation in vitrimers, with apparent activation energies reflecting the combined contributions of chemical exchange and segmental dynamics.^67^

In associative vitrimer systems (see Figure 3), new covalent bonds are formed before original bonds are cleaved, preserving network connectivity and preventing dissolution. In dissociative CANs, temporary bond cleavage leads to transient network fragmentation, formation of free chain segments, and possible dissolution prior to network reformation.^24,65,69^

**Figure 3:**
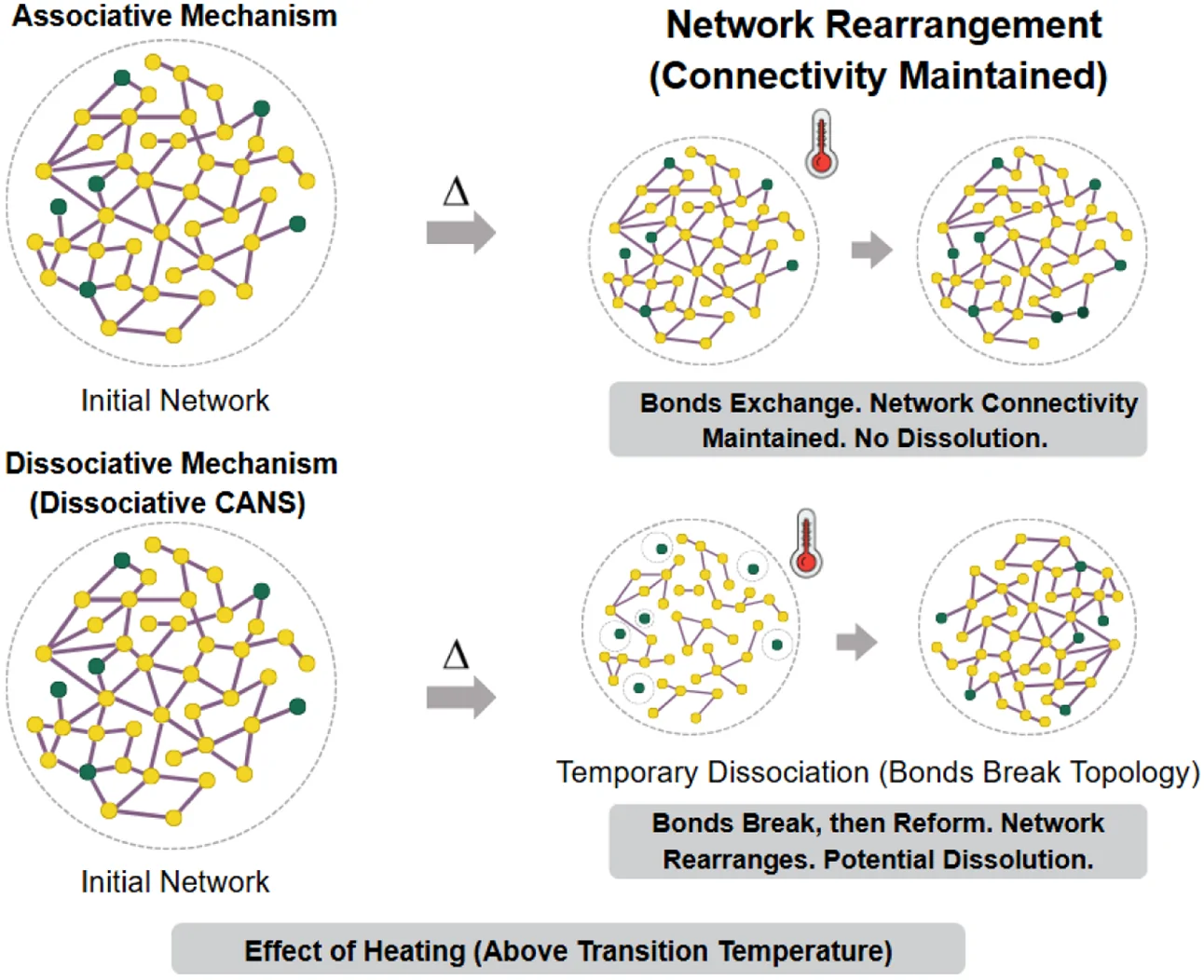
Comparison between associative vitrimer networks and dissociative covalent adaptable networks (CANs) upon thermal activation.

In semicrystalline PET, vitrimer behavior is inherently heterogeneous, as crystalline domains restrict chain mobility and limit the accessibility of ester groups to nucleophilic attack. Exchange reactions, therefore, occur predominantly in the amorphous phase, leading to broad relaxation spectra and nonuniform stress relaxation behavior. Several studies have demonstrated that increasing crystallinity results in longer relaxation times and higher apparent activation energies, underscoring the strong coupling between the physical morphology and chemical exchange kinetics in PET vitrimers.^20,70^

Furthermore, the associative transesterification mechanism enables PET vitrimers to undergo repeated reshaping, welding, and reprocessing cycles while preserving network integrity, offering a fundamentally different recycling paradigm compared with conventional thermoplastic remelting, where irreversible molecular weight loss dominates degradation.^71^

### 3.2 Catalyst Systems and Exchange Kinetics

Efficient transesterification in PET-based vitrimers is strongly dependent on the presence of catalysts capable of activating ester carbonyl groups and lowering the activation barrier for nucleophilic substitution. In the absence of catalysis, ester exchange reactions in polyesters proceed at impractically slow rates under typical processing conditions, rendering vitrimer behavior negligible within accessible temperature windows.^67^

#### 3.2.1 Metal-Based Catalysts

Metal-based catalysts represent the most widely employed catalytic systems for promoting transesterification in polyester vitrimers. Among these, zinc-based catalysts (e.g., Zn­(OAc)_2_ and ZnCl_2_) are particularly prominent due to their balanced catalytic activity, thermal stability, and comparatively low toxicity. Zinc ions act as Lewis acids, coordinating to the ester carbonyl oxygen, increasing the electrophilicity, and facilitating nucleophilic attack by hydroxyl groups.^42,44,71^

Tin-based catalysts, such as dibutyltin dilaurate (DBTDL) and stannous octoate (Sn­(Oct)_2_), exhibit higher catalytic efficiency and enable transesterification at lower temperatures,^25^ but raise concerns related to toxicity, catalyst migration, and long-term stability.^27^

#### 3.2.2 Organocatalytic and Epoxy–Ester Vitrimer Systems

In response to environmental and regulatory concerns associated with metal catalysts, organocatalytic systems have gained increasing attention. Organic bases and hydrogen-bonding catalysts can activate ester exchange by enhancing nucleophilicity or stabilizing transition states, albeit generally at the expense of higher activation temperatures.

Epoxy–ester vitrimer systems represent a particularly important class, as they combine permanent cross-link formation with the introduction of hydroxyl-rich environments that favor associative transesterification.^71^ Figure 4 illustrates the role of catalysts in controlling exchange kinetics and activation temperatures: (a) formation of epoxy–ester vitrimer networks through the reaction of multifunctional epoxides with tricarboxylic acids, generating β-hydroxyl ester groups that enable associative transesterification; and (b) transesterification reactions between diesters and diols promoted by thermal activation or metal-based catalysis (e.g., Sn­(Oct)_2_).

**Figure 4:**
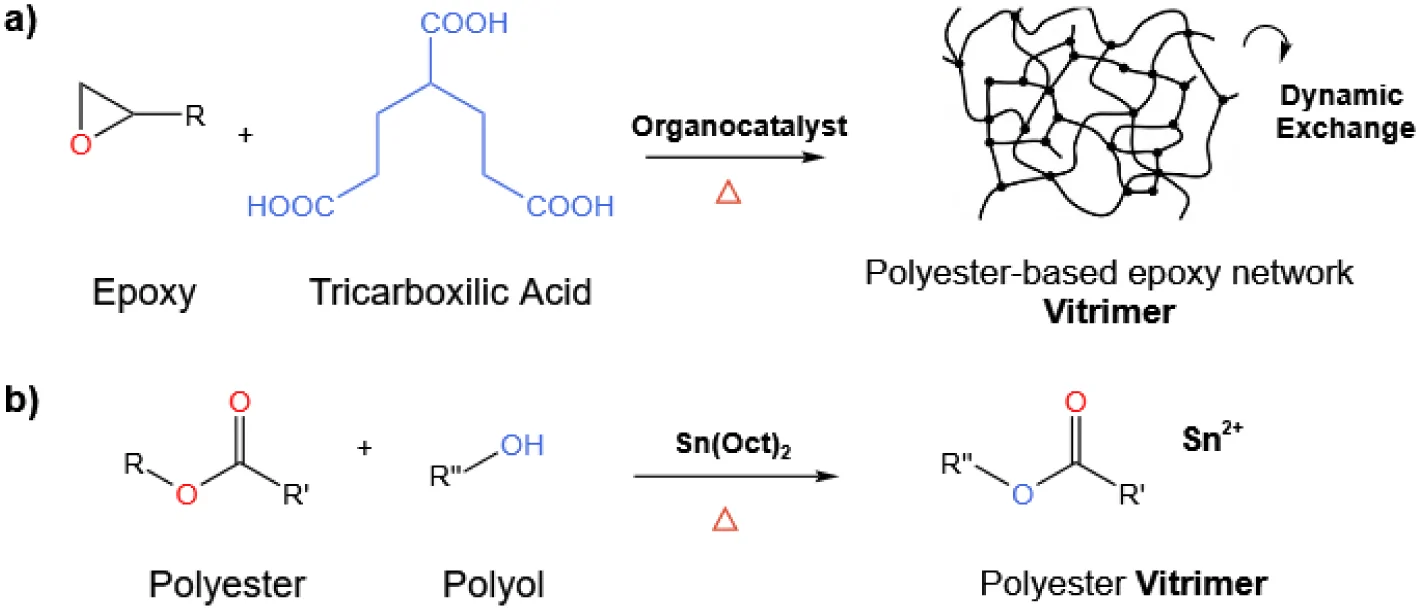
Representative chemical strategies for introducing dynamic ester exchange functionality in vitrimer networks. (a) Adapted with permission from Mao Chen, Hongwei Si, Huan Zhang, Lin Zhou, Yeping Wu, Lixian Song, Ming Kang, Xiu-Li Zhao; The Crucial Role in Controlling the Dynamic Properties of Polyester-Based Epoxy Vitrimers: The Density of Exchangeable Ester Bonds (υ). Macromolecules. 9 November 2021; 54(21): 10110–10117. https://doi.org/10.1021/acs.macromol.1c01289.^25^ Copyright 2021 American Chemical Society. (b) Adapted with permission from Suman Debnath, Swaraj Kaushal, Umaprasana Ojha; Catalyst-Free Partially Bio-Based Polyester Vitrimers. ACS Appl. Polym. Mater. 14 February 2020; 2(2): 1006–1013. https://doi.org/10.1021/acsapm.0c00016.^27^ Copyright 2020 American Chemical Society.

While traditional metal-based catalysts, such as zinc acetate (Zn­(OAc)_2_) and titanium­(IV) butoxide (Ti­(OBu)_4_), are extensively utilized to achieve high cross-linking efficiencies—with reported gel fractions reaching 84.5–95% in polylactic acid and polyethylene vitrimers—organocatalysts often demonstrate significantly higher catalytic activity in terms of bond-exchange kinetics.^19,28,45^ Quantitatively, strong Brønsted acid organocatalysts like ethylene glycol methacrylate phosphate (DMEP) can relax 63% of initial stress in just 15 min at 180 °C, whereas Zn­(OAc)_2_ requires 120 min under identical conditions.^34^

Furthermore, organocatalysts generally lower the activation energy (Ea) for transesterification more effectively than metal-based Lewis acids; for instance, strong protic acids exhibit Ea values between 49 and 67 kJ/mol, which is roughly half the 100–150 kJ/mol typically required for metal-catalyzed systems.^31^ Self-catalyzed polyester vitrimers employing internal tertiary amine groups also show enhanced activity, achieving relaxation times of 144 s at 170 °C, which is more than 20 times faster than systems where the amine catalyst is restricted to the cross-link points.^33^ Although metal catalysts provide robust mechanical performance and high thermal stability, organocatalytic approaches facilitate much faster processing at lower temperatures, with triflic acid predicted to be several orders of magnitude faster than zinc acetate at 140 °C (16 s versus approximately 10^4^ s).^31,45^

#### 3.2.3 Exchange Kinetics and Rheological Manifestation

The impact of catalyst type and concentration on exchange kinetics is typically quantified through stress relaxation and creep experiments, where the characteristic relaxation time (τ) decreases with increasing temperature and catalyst loading. In well-designed vitrimer systems, τ follows an Arrhenius-type relationship, enabling extraction of apparent activation energies that reflect both chemical exchange and polymer segmental mobility.^25,31,33,34,51^ However, excessive catalyst concentrations can be detrimental, promoting side reactions such as hydrolysis, chain scission, or uncontrolled transesterification, particularly under high-shear processing conditions, including reactive extrusion. Optimization of catalyst nature and loading is, therefore, critical to balance exchange efficiency, thermal stability, and long-term recyclability.^33,53,72^

## 4 Alternative and Hybrid Dynamic Chemistries in PET Vitrimer Systems

### 4.1 Rationale for Exploring Alternative and Hybrid Dynamic Chemistries

Although ester-based transesterification represents the most intrinsically compatible dynamic exchange mechanism for PET-based vitrimers, alternative and hybrid dynamic chemistries have been extensively explored in the broader vitrimer and covalent adaptable network (CAN) literature.^25,27,31,33,44^ The motivations for such approaches include the reduction of activation temperatures, acceleration of stress relaxation kinetics, introduction of additional functionalities, and mitigation of catalyst-related or processing constraints. However, the direct transfer of alternative dynamic chemistries to PET-based systems is inherently constrained by the chemical structure and semicrystalline nature of PET. Unlike amorphous or highly functionalized polymers, PET offers a limited density of reactive sites and exhibits restricted chain mobility in the solid and melt states. Consequently, alternative dynamic chemistries must be evaluated not only for their intrinsic exchange kinetics but also for chemical compatibility, moisture sensitivity, and robustness under melt-processing conditions.

### 4.2 Imine-Based Dynamic Networks

Imine (Schiff base) exchange reactions have been widely investigated because of their reversible covalent character and relatively low activation energies, enabling bond exchange under moderate thermal conditions.^73–76^ In PET-based materials, however, imine chemistry is not intrinsically compatible with the polyester backbone, as PET lacks amine or aldehyde functionalities.

Implementation, therefore, requires chemical modification or the introduction of external functional cross-linkers, which fundamentally alters the chemical identity of the material and often leads to heterogeneous network architectures. Moreover, imine bonds are highly susceptible to hydrolysis and moisture-induced degradation, a critical limitation for PET systems derived from postconsumer feedstocks, where complete moisture elimination is difficult to achieve.^76^ Consequently, imine-based dynamic networks are generally unsuitable for scalable PET vitrimer systems, particularly under melt-processing or reactive extrusion conditions, and are more appropriately restricted to model systems, coatings, or low-load applications.

### 4.3 Disulfide Exchange Reactions

Disulfide bonds enable dynamic exchange via radical- or thiolate-mediated mechanisms and have been successfully applied in self-healing polymers and CANs. Nevertheless, disulfide exchange is intrinsically dissociative, involving temporary bond cleavage prior to reformation, which leads to a transient loss of network connectivity.^11,77–80^

In PET-based systems, the incorporation of disulfide functionalities requires the use of sulfur-containing additives or cross-linkers that are not native to the polyester backbone. This introduces chemical discontinuity, increases susceptibility to creep, and compromises dimensional stability at elevated temperatures. The dissociative nature of disulfide exchange is fundamentally incompatible with the associative topological rearrangement required for true vitrimer behavior, particularly in semicrystalline polymers such as PET. Consequently, disulfide-based systems are poorly suited for PET vitrimer applications involving load-bearing performance or industrial melt-processing.^80–82^

### 4.4 Boronic Ester and Boronate-Based Dynamic Exchange

Boronic ester exchange reactions are characterized by fast kinetics and responsiveness to environmental stimuli, including moisture and pH. While advantageous in soft or stimuli-responsive materials, boronic ester chemistry presents significant challenges for PET-based vitrimers.^47–49^

Boronic esters are highly sensitive to water, undergoing rapid hydrolysis even at low moisture levels. This sensitivity is particularly problematic in PET recycling and reactive processing, where residual moisture is difficult to eliminate and water can be generated in situ during condensation-type reactions. The incorporation of boronic functionalities necessitates extensive chemical modification or blending with non-PET components, reducing compatibility with melt processing and undermining industrial relevance.^46,83,84^ As such, boronic ester-based networks are generally unsuitable for robust, scalable PET vitrimer systems.

### 4.5 Metal–Ligand Coordination Networks

Metal–ligand coordination interactions provide reversible bonding through coordination–decoordination processes and have been explored as dynamic motifs in polymer networks. In PET-based systems, however, coordination interactions are typically insufficient to sustain vitrimer behavior as the primary exchange mechanism, owing to their comparatively lower bond strength and sensitivity to competing ligands, solvents, or moisture.

Recent advancements in multifunctional elastomeric networks have demonstrated the successful use of metal–ligand coordination to achieve self-repairing properties. In particular, Biswas et al.^85^ and Das et al.^86^ employed this chemistry in carboxylated nitrile butadiene rubber (XNBR) by grafting 1-(3-aminopropyl)­imidazole (API) onto the polymer backbone and cross-linking it with zinc oxide (ZnO). These systems exploit the reversible coordination between Zn^2+^ ions and API ligands to attain high self-healing efficiencies (up to 78.2% at 45 °C or at room temperature) and complete recyclability, while maintaining performance in applications such as electromagnetic interference (EMI) shielding and energy harvesting. However, the successful implementation of this approach in elastomers contrasts with the challenges encountered in PET-based vitrimer systems.

While coordination bonds remain stable under the moderate conditions used for XNBR, the high temperatures and intense shear stresses associated with PET reactive extrusion (RE) can promote their uncontrolled dissociation, leading to loss of mechanical integrity. Therefore, in PET systems, metal–ligand interactions are more suitably employed as auxiliary dynamic motifs to tune relaxation behavior or impart secondary functionalities rather than as the primary mechanism for network topology rearrangement. Metal–ligand interactions as auxiliary dynamic interactions are capable of modulating relaxation behavior or imparting secondary functionality when combined with ester-based exchange mechanisms.^42,43^

### 4.6 Hybrid Dynamic Chemistries: Synergistic and Antagonistic Effects

Hybrid vitrimer systems combining ester-based transesterification with secondary dynamic interactions have been proposed as strategies to tailor the relaxation kinetics, processing windows, and mechanical response. In such systems, transesterification governs permanent network rearrangement, while secondary interactions (e.g., coordination or hydrogen bonding) provide additional degrees of freedom.^87–90^

Hybridization introduces significant design complexity, particularly in PET-based systems where crystallinity already constrains exchange reactions spatially. Competing dynamic pathways may lead to antagonistic effects, including uncontrolled stress relaxation, excessive creep, or enhanced susceptibility to thermal and hydrolytic degradation. Therefore, hybrid dynamic chemistries must be implemented with caution, ensuring that ester-based transesterification remains the dominant mechanism controlling the network topology and recyclability.^46–48^

### 4.7 Critical Comparison with Ester-Based Transesterification

When critically evaluated in the specific context of PET, ester-based transesterification remains the most intrinsically compatible and practically viable dynamic exchange mechanism for vitrimers. Its compatibility with the polyester backbone, associative exchange mechanism, and tolerance to melt processing conditions distinguish it from imine-, disulfide-, boronic-, and coordination-based alternatives. The comparison between these exchange mechanisms is summarized in Figure 5.

**Figure 5:**
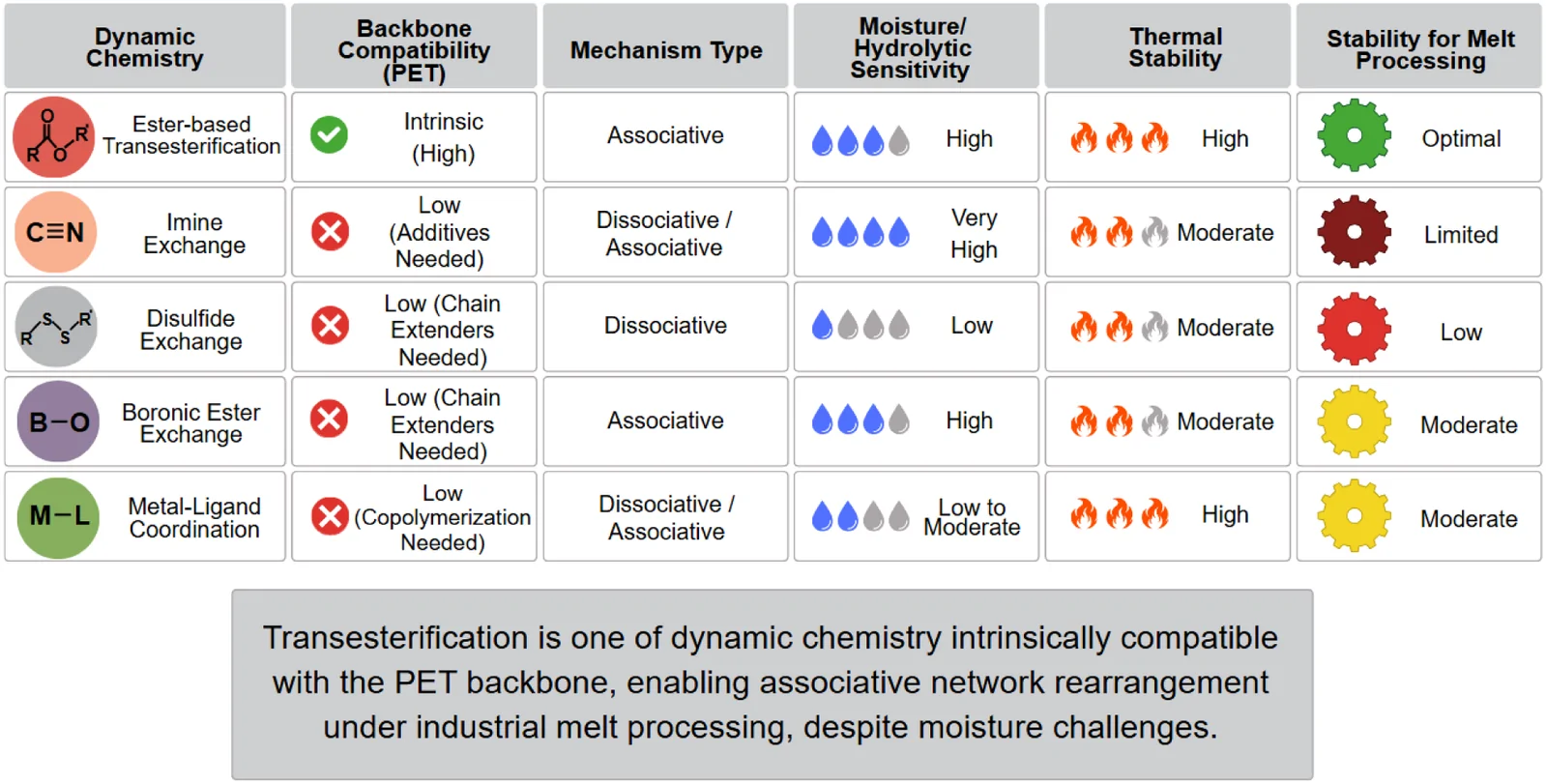
Comparative overview of dynamic chemistries investigated for dynamic PET-based networks.

The above statement does not imply that ester-based transesterification is free of limitations. Depending on the reaction pathway employed, particularly those involving condensation reactions or acid-mediated processes, transesterification may be accompanied by in situ generation of water, which can promote hydrolytic chain scission and molecular weight reduction in PET.^39,40,90,91^

Despite these process-related challenges, ester-based transesterification remains the dynamic exchange mechanism intrinsically compatible with the PET backbone and capable of sustaining associative vitrimer behavior under melt-processing and reactive extrusion conditions, provided that reaction design, catalyst selection, and moisture management are carefully controlled.

### 4.8 Implications and Transition to Processing Considerations

The critical assessment presented in this section demonstrates that chemical compatibility alone is insufficient to guarantee viable PET vitrimers. The interplay among dynamic chemistry, moisture generation, crystallinity, and processing conditions must be considered within an integrated design framework.

These considerations directly motivate the following section, which examines the interaction between vitrimer chemistry and PET’s semicrystalline structure, and subsequently examines the challenges associated with processing routes and reactive extrusion, where moisture control and thermal stability become decisive factors.

## 5 Interaction between Vitrimer Chemistry and PET’s Semicrystalline Structure

### 5.1 Semicrystalline Nature of PET and Its Implications for Dynamic Exchange

PET is a semicrystalline polyester, typically exhibiting crystallinity levels ranging from approximately 25% to 45% depending on molecular weight, thermal history, and processing conditions. This heterogeneous morphology, consisting of crystalline lamellae embedded within an amorphous matrix, fundamentally distinguishes PET from amorphous polyesters frequently employed in vitrimer model systems.^92–94^

In the context of vitrimer chemistry, this semicrystalline morphology imposes intrinsic spatial constraints on the dynamic exchange reactions. Ester-based transesterification, as well as any alternative dynamic mechanism, relies on the segmental mobility and local accessibility of reactive groups. Crystalline domains, characterized by tightly packed chain conformations and reduced free volume, are effectively chemically inactive regions with respect to bond exchange reactions. As a consequence, vitrimer behavior in PET is inherently heterogeneous, with dynamic exchange reactions occurring predominantly and often exclusively within the amorphous phase. As Denissen et al.^67^ explained, without sufficient segmental mobility, the reactive functional groups are unable to move and encounter one another, meaning that no exchange reactions can occur regardless of the intrinsic chemical reactivity.

Valdez et al.^95^ studied the nonisothermal crystallization kinetics of polyethylene-derived vitrimers to understand the interplay between cross-linking and crystal growth. These studies focused on how dynamic networks and transesterification catalysts influence the formation of crystalline regions under cooling rates that are relevant to industrial processing. Valdez and collaborators demonstrated this behavior by showing that whereas cross-link formation suppresses crystal growth rates, the presence of catalysts can act as nucleating agents that significantly modify the crystallization activation energy.

### 5.2 Confinement of Dynamic Exchange to the Amorphous Phase

Experimental and theoretical studies consistently demonstrate that dynamic bond exchange in semicrystalline vitrimers is spatially confined to amorphous regions,^96,97^ where chain mobility is sufficient to enable nucleophilic attack and bond rearrangement. In PET-based vitrimer systems, ester exchange reactions are therefore localized within the amorphous interlamellar phase, leading to a decoupling between chemical exchange kinetics and the overall macroscopic morphology.

This confinement has several critical consequences:

- The effective concentration of exchangeable bonds is reduced relative to that of fully amorphous systems;
- Stress relaxation proceeds via localized rearrangements, resulting in broad relaxation-time distributions;
- Complete topological equilibrium of the network may be kinetically inaccessible under practical temperatures and time scales.

These effects collectively explain why PET vitrimers typically exhibit slower stress relaxation and higher apparent activation energies compared with vitrimer systems based on amorphous polyesters, even when similar catalysts and exchange chemistries are employed.^98^

Figure 6 illustrates the heterogeneous morphology of PET, consisting of an amorphous matrix embedded within crystalline lamellae. Ester-based transesterification reactions occur predominantly within the amorphous regions, where segmental mobility and accessibility of reactive groups are sufficient to enable bond exchange. Crystalline domains act as chemically inactive regions, restricting chain mobility and leading to spatially confined exchange, heterogeneous stress relaxation behavior, and increased apparent activation energies in PET-based vitrimer systems.

**Figure 6:**
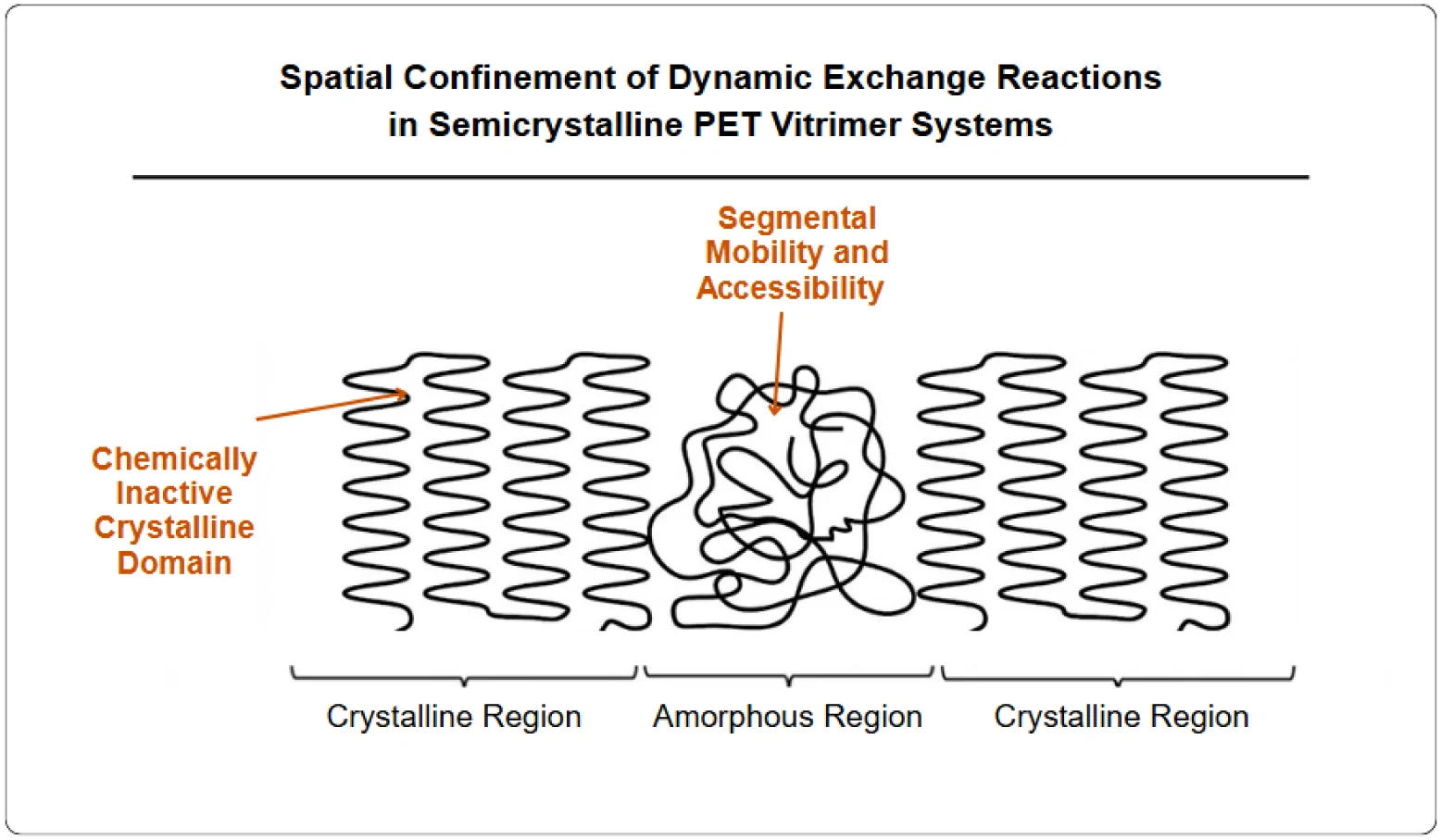
Spatial confinement of dynamic exchange reactions in semicrystalline PET vitrimer systems.

### 5.3 Influence of Crystallinity on Stress Relaxation and Activation Energy

Stress relaxation experiments provide a direct macroscopic manifestation of the interaction between vitrimer chemistry and semicrystalline morphology. In PET-based vitrimers, relaxation times (τ) systematically increase with increasing crystallinity, reflecting the reduced fraction of dynamically active material.^92,99^

From an Arrhenius perspective, the apparent activation energy (Ea) extracted from relaxation measurements encompasses not only the intrinsic chemical barrier associated with transesterification but also the energetic penalty associated with activating segmental motion in constrained amorphous regions adjacent to crystalline lamellae.^25,37,84,95,99^

As the crystallinity increases, this additional energetic contribution becomes more pronounced, leading to higher apparent Ea values and a stronger temperature dependence of stress relaxation. Consequently, Ea in semicrystalline PET vitrimers must be interpreted as a convoluted parameter reflecting both chemical exchange and morphological constraints rather than as a purely chemical descriptor.

### 5.4 Effect of Thermal History and Processing-Induced Morphology

The semicrystalline structure of PET is highly sensitive to the thermal history, including the cooling rate, annealing conditions, and processing temperature. As a result, the vitrimer response of PET is not an intrinsic material constant but a processing-dependent property.

Rapid cooling from the melt suppresses crystallization, increasing the amorphous fraction and facilitating dynamic exchange reactions. Conversely, slow cooling or postprocessing annealing enhances crystallinity, restricting exchange kinetics and slowing stress relaxation.^93,100,101^

The processing routes that promote crystallization during or after vitrimer network formation can effectively “lock in” the network topology, limiting subsequent rearrangement. This observation highlights the necessity of synchronizing chemical vitrimerization and morphological development, particularly in processing strategies involving reactive extrusion.

### 5.5 Interplay between Crystallinity, Hydrolysis, and Molecular Weight Evolution

In PET-based vitrimers, the interaction between semicrystallinity and chemical stability is further complicated by hydrolytic sensitivity.^102^ Water, whether introduced as residual moisture or generated in situ during reactive processes, preferentially attacks ester bonds in amorphous regions, where accessibility is highest.

This selective hydrolysis leads to localized chain scission within the dynamically active phase, altering molecular weight distribution, increasing the concentration of chain ends, and potentially accelerating subsequent transesterification reactions. While this may transiently enhance exchange kinetics, it ultimately compromises the mechanical integrity and long-term recyclability. Crystalline domains, by contrast, offer partial protection against hydrolysis, reinforcing the heterogeneous nature of the degradation and exchange processes in PET vitrimer systems.

### 5.6 Consequences for Vitrimer Design and Performance

The strong coupling between vitrimer chemistry and semicrystalline morphology in PET leads to several critical design implications:

- Catalyst selection and loading must account for restricted chain mobility, as overly aggressive catalysis may promote degradation rather than uniform exchange.
- Target crystallinity levels must be carefully optimized, balancing mechanical performance against the need for sufficient amorphous content to enable stress relaxation;
- Reported vitrimer metrics (τ, Ea, reprocessability) must be interpreted in the context of morphology and thermal history, rather than directly compared with amorphous vitrimer systems.

Failure to consider these aspects can lead to misleading conclusions regarding vitrimer efficiency, scalability, and long-term performance.

Lv et al.^103^ investigated the quantitative relationship between crystallinity and key physical properties, particularly the intrinsic thermal conductivity of ethylene-based vitrimers. Their work focused on tracking the evolution of polymer chain conformations from a disordered amorphous state to an ordered semicrystalline, polycrystalline structure during aging. The authors demonstrated that bond exchange reactions that occur within the amorphous phase facilitate the rearrangement of ethylene segments, promoting crystallization and resulting in an approximately 10-fold increase in thermal conductivity as the degree of crystallinity increases.

### 5.7 Implications for Processing and Scalability

The insights discussed in this section demonstrate that PET vitrimer performance is inseparable from morphology control.^96,97^ Any processing strategy aimed at industrial scalability must therefore integrate:

- Control of crystallization kinetics,
- Management of thermal history,
- Preservation of molecular weight,
- Mitigation of hydrolytic degradation

These considerations directly motivate the following section, which examines processing routes and reactive extrusion of PET vitrimers, where the combined effects of heat, shear, residence time, and moisture critically determine the final material properties.

## 6 Processing Routes and Reactive Extrusion of PET-Based Systems

### 6.1 From Laboratory Synthesis to Scalable Processing

Nettles et al.^104^ studied reactive extrusion (RE) as a solvent-free platform for rapid polymer deconstruction and upcycling to value-added materials. These studies focused on the implementation of in situ chemorheological characterization techniques, such as near-infrared spectroscopy, to monitor structural transformations during processing. They demonstrated this behavior by showcasing how RE leverages legacy industrial infrastructure to enable precise control over molecular weight and branching through mechanochemical reactions.

The translation of PET-based system concepts from laboratory-scale demonstrations to industrially relevant materials is fundamentally governed by processing route selection.^105,106^ While batch processing methods (e.g., melt mixing in internal mixers, compression molding, or solution-based approaches) are valuable for mechanistic studies and proof-of-concept demonstrations, they are inherently limited in terms of throughput, reproducibility, and scalability.

For PET-based systems, these limitations are particularly pronounced because of the high melting temperature, semicrystalline nature, and hydrolytic sensitivity of PET.^107^ As a result, RE has emerged as the most relevant processing strategy for scalable dynamic PET-based network production, enabling continuous processing, precise control of the residence time, and direct integration into existing polymer recycling and compounding infrastructure.^28,57,108^

### 6.2 Batch Processing Routes: Advantages and Intrinsic Limitations

Batch processing routes are frequently employed in the early stages of PET-based system development, allowing precise control over formulation variables, catalyst content, and thermal protocols. Such approaches facilitate fundamental studies of exchange mechanisms, stress relaxation behavior, and structure–property relationships.^109,110^

However, batch processing is poorly suited to dynamic PET-based network systems intended for industrial deployment. Limitations include:

- Limited control over shear history and residence time.
- Difficulty in achieving homogeneous dispersion of catalysts and cross-linkers in viscous PET melts;
- Restricted ability to remove moisture and volatile byproducts;
- Poor representation of industrial melt-processing conditions.

In summary, results obtained under batch conditions often overestimate vitrimer efficiency and reprocessability, particularly when they are translated into continuous processing environments.

The contrast between laboratory-scale batch processing methods (e.g., internal mixing and compression molding) and reactive extrusion in terms of control over shear, residence time, moisture removal, scalability, and compatibility with industrial PET recycling infrastructure is shown in Figure 7. Reactive extrusion offers superior process control and scalability, whereas batch processing is better suited for mechanistic studies and proof-of-concept demonstrations.

**Figure 7:**
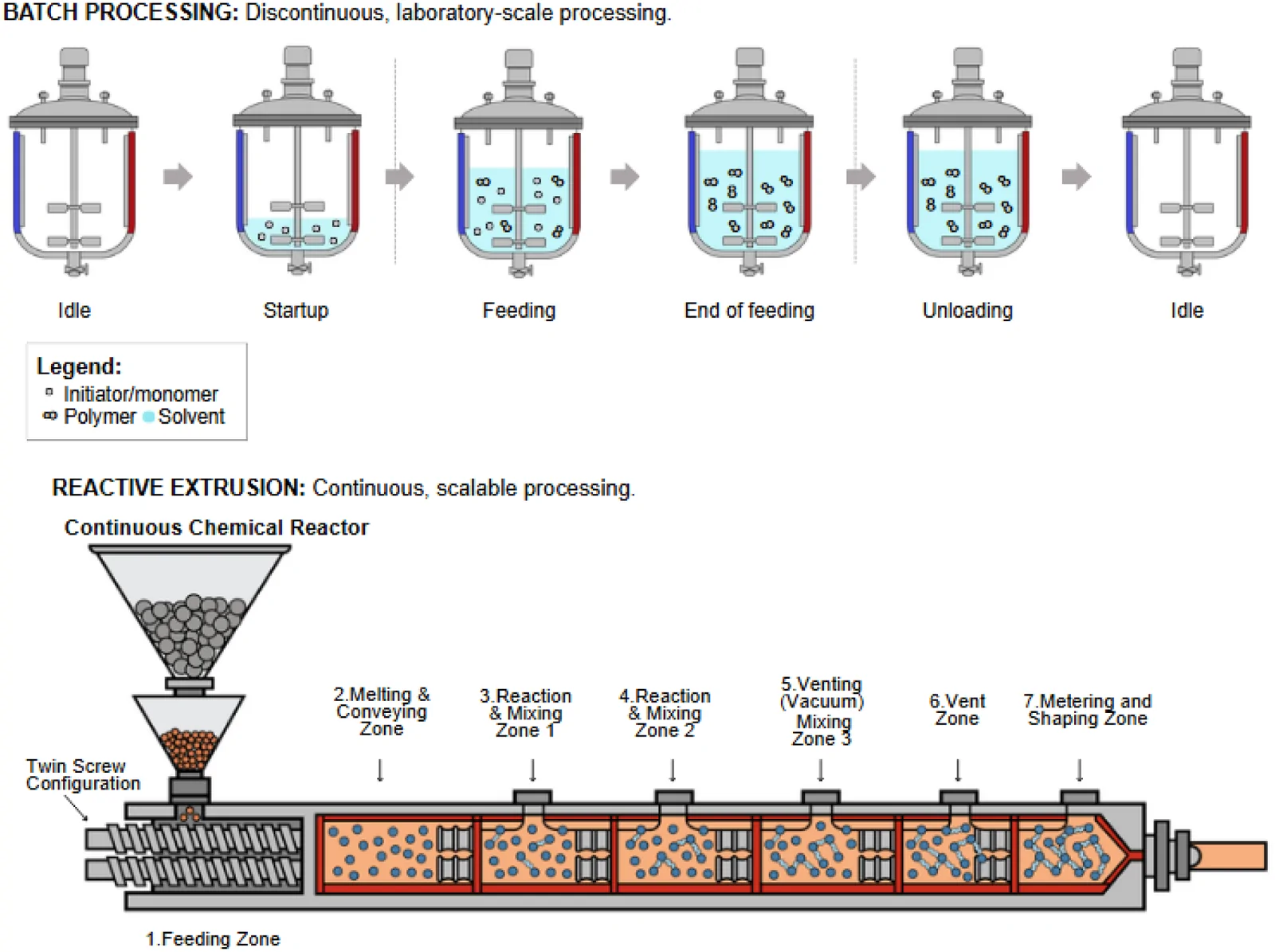
Batch processing and reactive extrusion routes for PET vitrimer systems.

The choice between batch and continuous processing significantly influences the final properties of vitrimerized polymers, primarily due to varying shear stresses and residence times. Discontinuous batch processing, such as reactive microcompounding, ensures a homogeneous mixture of vitrimerizing agents through a recirculation channel that returns the melt to the screws. In contrast, continuous reactive extrusion (CRE) utilizes modular screw profiles with distinct mixing zones that generate high local shear stress. While CRE is more scalable, research on polybutylene terephthalate (PBT) indicates that higher screw rotation speeds (100–200 rpm) favor chain scission over branching, which can hinder gelation and prevent the formation of a three-dimensional network. For instance, while batch microcompounding achieved a cross-link density (νc) of 2, continuous extrusion at the same temperature but at 200 rpm resulted in a density of only 0.05, effectively transitioning the material from a viscoelastic solid back to a viscoelastic liquid.^106^

### 6.3 Reactive Extrusion as a Platform for the Production of PET-Based Systems

Reactive extrusion offers a unique combination of thermal, mechanical, and chemical control, making it the most promising route for the scalable production of PET-based systems. In RE, PET is processed in the melt state under controlled shear, temperature, and residence time, while dynamic cross-linkers and catalysts are introduced in situ.^4,57,108,111^

For dynamic covalent systems based on ester exchange reactions, RE enables simultaneous:

- Network formation and dynamic bond introduction;
- Control of molecular-weight evolution;
- Dispersion of catalysts and additives;
- They were shaped into pellets, sheets, or profiles.

RE closely reflects the processing realities of PET recycling, where postconsumer feedstocks, variable molecular weights, and residual contaminants are unavoidable.

### 6.4 Residence Time, Shear, and Exchange–Degradation Competition

A defining challenge in the reactive extrusion of dynamic PET-based networks is the competition between dynamic bond exchange and irreversible degradation mechanisms. Ester-based transesterification requires sufficient thermal activation and residence time to proceed effectively, yet prolonged exposure to high temperature and shear accelerates hydrolytic and thermomechanical degradation.^13,106,112–114^

In PET-based systems, this competition manifests as a narrow process window, in which:

- The residence time must be long enough to enable network rearrangement;
- Shear must be sufficient to promote mixing and catalyst dispersion.
- The temperature must activate exchange reactions without inducing excessive chain scission.

Failure to balance these parameters can result in apparent dynamic exchange behavior driven by molecular weight reduction rather than genuine associative exchange, leading to misleading interpretations of stress relaxation and reprocessability.

Figure 8 is a diagram that illustrates the competition between ester-based associative exchange reactions and irreversible degradation mechanisms as a function of temperature, shear, and residence time. With insufficient thermal or mechanical activation, exchange reactions are too slow to induce effective vitrimer behavior. At excessive temperatures or prolonged residence times, hydrolytic and thermomechanical degradation dominate, leading to molecular weight reduction. An optimal processing window exists in which dynamic exchange is activated while degradation is minimized. Moisture content, catalyst selection, and feedstock quality (virgin versus recycled PET) critically influence the width and position of this window.

**Figure 8:**
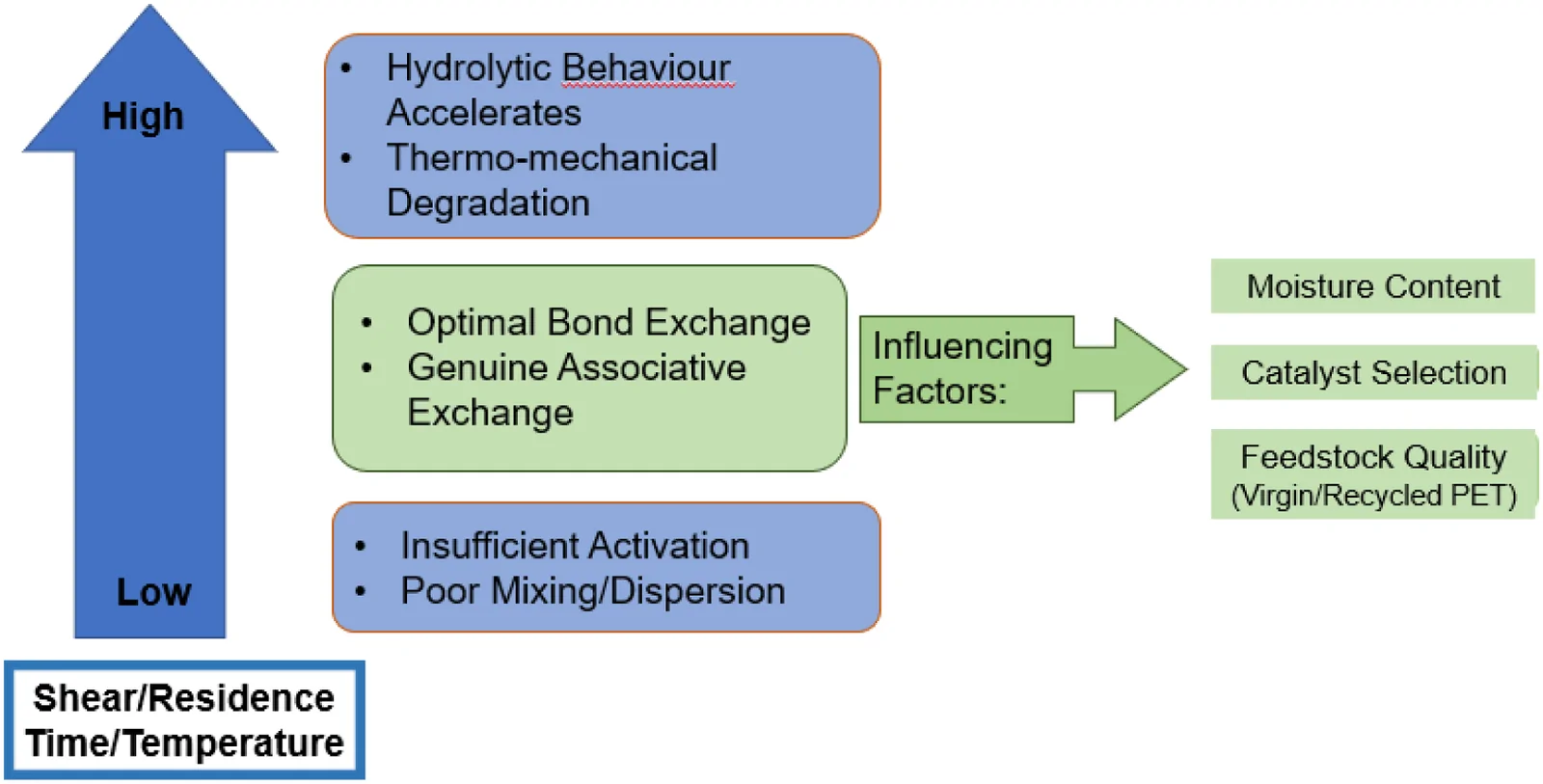
Schematic processing window for dynamic PET-based systems under reactive extrusion.

### 6.5 Moisture Management and Volatile Byproduct Control

Moisture management is a critical determinant of success in the reactive extrusion of dynamic PET-based systems. Water may be present as residual moisture in recycled PET, absorbed during handling, or generated in situ during condensation-type reactions involving carboxylic acids.

Even trace amounts of water can catalyze hydrolytic chain scission, particularly in the amorphous phase, where dynamic exchange reactions occur. In RE, where continuous venting is limited and residence times are short, moisture accumulation can rapidly compromise the molecular weight and mechanical integrity.^102^

Effective mitigation strategies include:

- Rigorous predrying of PET feedstocks;
- Selection of exchange pathways that minimize water generation;
- Incorporation of vacuum venting or devolatilisation zones;
- Careful catalyst selection avoids excessive hydrolytic activity.

These considerations highlight that the dynamic bond-exchange chemistry and processing design cannot be decoupled.

### 6.6 Influence of Reactive Extrusion on Morphology and Dynamic Exchange Performance

Reactive extrusion not only governs chemical exchange but also strongly influences PET morphology, particularly crystallization behavior: shear, temperature gradients, and cooling rates during extrusion affect the development of crystalline lamellae and the distribution of amorphous regions.^96,99^

As discussed in Section 5, dynamic exchange reactions are confined to amorphous domains. Consequently, processing-induced changes in crystallinity directly impact the vitrimer performance. High shear and rapid cooling may suppress crystallization, enhancing exchange kinetics, whereas slow cooling or postextrusion annealing can increase crystallinity and restrict network rearrangement.

The reactive extrusion parameters indirectly modulate the vitrimer efficiency through morphological control, reinforcing the need for integrated process design.

### 6.7 Reactive Extrusion of Recycled PET: Opportunities and Constraints

The integration of vitrimer chemistry into recycled PET streams represents a compelling opportunity to upgrade material performance while preserving circularity. Recycled PET typically exhibits a reduced molecular weight and increased end-group concentration, which can facilitate dynamic exchange reactions.

However, recycled feedstocks also introduce challenges, including:

- Variable moisture content;
- Contaminants and legacy additives;
- Broader molecular-weight distribution.

Reactive extrusion of PET vitrimers must therefore accommodate feedstock variability, requiring robust processing windows and a tolerance for compositional fluctuations. Strategies relying on a narrow stoichiometric balance or on highly sensitive catalysts may prove unsuitable under such conditions.

### 6.8 Scalability, Industrial Relevance, and Process Realism

The effectiveness of chemical modification is evidenced by significant quantitative enhancements in material performance and reaction yields across various polyester systems.^4,8,51^ Fabrizio et al.^8^ demonstrated this pattern through the upcycling of poly­(ethylene terephthalate) (PET) from food trays, where reactive melt processing with 1.36 wt % diglycidyl ether of bisphenol A (DGEBA) and 0.3 wt % zinc catalyst at 250 °C produced a vitrimeric network with an initial cross-linked gel fraction of 33–44%, which reached up to 75% at higher additive concentrations. This modification resulted in a 2-fold increase in mechanical strength, with tensile strength rising from 23.5 to 54.8 MPa and elongation at break improving from 1.2% to 6.0%. Similarly, Maaz et al.^4^ demonstrated this behavior in the synthesis of polyethylene (PE) vitrimers, achieving quantitative grafting yields of approximately 101% when using optimized protocols that involved premixing at 140 °C prior to reaction at 170 °C, yielding gel contents between 35% and 47%. Furthermore, Wu et al.^51^ focused on catalyst-free PET vitrimers fabricated via melt reaction with 1.2 wt % N,N′-tetraglycidyldiaminodiphenylmethane (TGDDM) at 260 °C, which attained a peak gel fraction of 76.3% within several minutes.

Despite significant progress at the laboratory scale, few dynamic PET-based network systems have been validated under truly industrially relevant reactive extrusion conditions. Many studies employ low screw speeds, extended residence times, or idealized feedstocks that do not reflect industrial practice.^56,115^

For PET vitrimers to transition beyond academic demonstrations, future work must prioritize:

- Validation under high-throughput RE conditions;
- Assessment of long-term stability over multiple processing cycles.
- Evaluation of mechanical-performance retention after repeated extrusion.
- Compatibility with the existing PET recycling infrastructure.

Only by addressing these factors can dynamic PET-based systems achieve a meaningful industrial impact.

### 6.9 Implications for Design Principles and Outlook

The analysis presented in this section demonstrates that processing is a central design variable in dynamic PET-based systems rather than a secondary consideration. Reactive extrusion imposes simultaneous constraints on the chemistry, morphology, and stability, requiring a holistic and realistic design approach.

These insights directly motivate the final section of this Review, which critically examines the current limitations, technological gaps, and market-related challenges facing PET vitrimer systems and outlines realistic pathways toward scalable implementation.

## 7 Main Achievements

- This Review demonstrates that ester-based transesterification is the dynamic covalent exchange mechanism intrinsically compatible with PET under melt-processing conditions, while alternative chemistries systematically fail to meet the chemical, thermal, and moisture-tolerance constraints imposed by semicrystalline PET and industrial recycling environments.
- It establishes that vitrimer behavior in PET is fundamentally morphology-limited rather than chemistry-limited, as dynamic exchange reactions are spatially confined to the amorphous phase, leading to heterogeneous relaxation dynamics and elevated apparent activation energies.
- The analysis further clarifies that macroscopic stress relaxation alone is insufficient to validate vitrimer performance, since apparent flow may originate from irreversible molecular-weight reduction rather than genuine associative exchange. Reliable vitrimer assessment therefore requires the combined evaluation of the relaxation kinetics, molecular-weight retention, and reprocessability.
- The review identifies reactive extrusion as a defining processing platform and moisture management as a first-order design variable, since only a narrow processing window enables effective bond exchange while suppressing hydrolytic and thermomechanical degradation. Together, these insights provide practical design rules for the scalable upcycling of PET to high-performance vitrimer materials.

## 8 Conclusion

This Review demonstrates that the performance and scalability of PET-based vitrimer systems are governed by a strong coupling between dynamic covalent chemistry, semicrystalline morphology, and processing realism. Among the dynamic exchange mechanisms reported, ester-based transesterification remains the chemistry intrinsically compatible with the PET backbone under melt processing conditions, while alternative approaches are fundamentally limited by chemical incompatibility, moisture sensitivity, or insufficient thermal robustness.

A key conclusion is that vitrimer behavior in PET is morphology-limited rather than chemistry-limited, as dynamic exchange reactions are spatially confined to the amorphous phase of a semicrystalline matrix. This confinement leads to heterogeneous relaxation dynamics and elevated apparent activation energies, requiring careful interpretation of vitrimer metrics and precluding a direct comparison with amorphous vitrimer systems.

From a processing standpoint, reactive extrusion is identified as a defining platform for the development of industrially relevant PET vitrimers’ development. Effective vitrimerization is restricted to a narrow processing window in which associative exchange must be activated while suppressing thermomechanical and hydrolytic degradation, with moisture control emerging as a first-order design parameter.

Overall, this Review highlights that successful PET vitrimer design cannot rely on chemistry alone but must integrate morphology control and realistic processing constraints. These insights provide a clear framework to guide future developments toward scalable, high-performance PET vitrimer systems aligned with circular polymer economy strategies.

## #

Department of Materials Engineering, Federal University of Campina Grande, Campina Grande, PB, Brazil

## References

[1] Market Growth Reports Polyethylene Terephthalate (PET) Market Report; Market Growth Reports; 2034. https://www.marketgrowthreports.com/market-reports/polyethylene-terephthalate-pet-market-118314?utm_source=chatgpt.com. accessed 05 Febuary 2026.

[2] Kaur G., Kumar P., Sonne C. (2024). Properties
and Biomedical Perspective
on Vitrimers – Challenges & Opportunities. RSC Appl. Interfaces.

[3] Amundarain I., López-Montenegro S., Fulgencio-Medrano L., Leivar J., Iruskieta A., Asueta A., Miguel-Fernández R., Arnaiz S., Pereda-Ayo B. (2024). Improving the Sustainability of Catalytic
Glycolysis of Complex PET Waste through Bio-Solvolysis. Polymer.

[4] Maaz M., Riba-Bremerch A., Guibert C., Van Zee N. J., Nicolaÿ R. (2021). Synthesis
of Polyethylene Vitrimers in a Single Step: Consequences of Graft
Structure, Reactive Extrusion Conditions, and Processing Aids. Macromolecules.

[5] Polyethylene Terephthalate Market Size, Share and Outlook - Growth Analysis Report and Forecast Trends (2026-2035); 2026. https://www.expertmarketresearch.com/reports/polyethylene-terephthalate-market?utm_source=chatgpt.com.

[6] Ng K. W. J., Lim J. S. K., Gupta N., Dong B. X., Hu C. P., Hu J., Hu X. M. (2023). A Facile
Alternative Strategy of Upcycling Mixed Plastic
Waste into Vitrimers. Commun. Chem..

[7] Kuete M. A., Van Velthem P., Ballout W., Nysten B., Devaux J., Ndikontar M. K., Pardoen T., Bailly C. (2022). Integrated Approach
to Eco-Friendly Thermoplastic Composites Based on Chemically Recycled
PET Co-Polymers Reinforced with Treated Banana Fibres. Polymers.

[8] Fabrizio L., Arrigo R., Scrivani M. T., Monti M., Fina A. (2023). Upcycling
of PET from Recycled Food Packaging Trays via Vitrimers Chemistry. Polymer.

[9] Mohammadi S., Bouldo M. G., Enayati M. (2023). Controlled Glycolysis of Poly­(Ethylene
Terephthalate) to Oligomers under Microwave Irradiation Using Antimony­(III)
Oxide. ACS Appl. Polym. Mater..

[10] Saalwächter K., Soman B., Evans C. M. (2023). Crystallinity
and Perfection in Ethylene
Vitrimers Probed by Combined Calorimetry, Scattering, and Time-Domain
NMR. Front. Soft Matter..

[11] Shi Q., Jin C., Chen Z., An L., Wang T. (2023). On the Welding
of Vitrimers:
Chemistry, Mechanics and Applications. Adv.
Funct. Mater..

[12] Trinh B., Owen P., VanderHeide A., Gupta A., Mekonnen T. H. (2023). Recyclable
and Self-Healing Natural Rubber Vitrimers from Anhydride-Epoxy Exchangeable
Covalent Bonds. ACS Appl. Polym. Mater..

[13] Soenen W., Poilvet G., Sekar A., Dul S., Hervieu C., Gaan S. (2025). Reactive Extrusion of Vitrimers: A Short Review. Sustain. Mater. Technol..

[14] Cuminet F., Caillol S., Dantras É., Leclerc É., Ladmiral V. (2021). Neighboring
Group Participation and Internal Catalysis Effects on Exchangeable
Covalent Bonds: Application to the Thriving Field of Vitrimer Chemistry. Macromolecules.

[15] Qiu J., Ma S., Wang S., Tang Z., Li Q., Tian A., Xu X., Wang B., Lu N., Zhu J. (2021). Upcycling of Polyethylene
Terephthalate to Continuously Reprocessable Vitrimers through Reactive
Extrusion. Macromolecules.

[16] Jia D. Z., Li L., Yang Y., Zhang J., Yang T. B., Lin H., Huang H. D., Zhong G. J., Li Z. M. (2025). Recycling Thermoplastic
Polyester with Dynamic Covalent Networks. Macromolecules.

[17] Montarnal D., Capelot M., Tournilhac F., Leibler L. (2011). Silica-like Malleable
Materials from Permanent Organic Networks. Science.

[18] Wang S., Liu Y., Zhao H., Lu C., Fu Y., Zhao S., Dong Y., Li J., Pang C. (2025). Catalyst-Free Covalent
Adaptable Polyester Networks Based on Dissociative Transesterification
Chemistry. Macromolecules.

[19] Rizzo G., Saitta L., Dattilo S., Tosto C., Pergolizzi E., Ivankovic A., Cicala G. (2023). Thermomechanical Characterization
of an Unsaturated Polyester Vitrimer Synthesized Using a Titanium
Transesterification Catalyst. ACS Appl. Polym.
Mater..

[20] Capelot M., Montarnal D., Tournilhac F., Leibler L. (2012). Metal-Catalyzed Transesterification
for Healing and Assembling of Thermosets. J.
Am. Chem. Soc..

[21] Amin O., Ramzy A., Abou-Elmagd W. S. I., Abdellatif M. H. (2025). Enhanced
Healability and Weldability Properties of Vitrimers. Polym. Eng. Sci..

[22] Cornellà A. C., Furia F., Van Assche G., Brancart J. (2024). Controlling the Relaxation
Dynamics of Polymer Networks by Combining Associative and Dissociative
Dynamic Covalent Bonds. Adv. Mater..

[23] Hayashi M., Obara H., Miwa Y. (2021). Design and Basic Properties
of Polyester
Vitrimers Combined with an Ionomer Concept. Mol. Syst. Des. Eng..

[24] Schaufelberger, F. ; Timmer, B. J. J. ; Ramström, O. Principles of Dynamic Covalent Chemistry Dynamic Covalent Chemistry John Wiley & Sons Ltd. 2017 1–30 10.1002/9781119075738.CH1

[25] Chen M., Si H., Zhang H., Zhou L., Wu Y., Song L., Kang M., Zhao X. L. (2021). The Crucial Role in Controlling the
Dynamic Properties of Polyester-LBased Epoxy Vitrimers: The Density
of Exchangeable Ester Bonds (υ). Macromolecules.

[26] Concilio M., Sulley G. S., Vidal F., Brown S., Williams C. K. (2025). Precise
Carboxylic Acid-Functionalized Polyesters in Reprocessable Vitrimers. J. Am. Chem. Soc..

[27] Debnath S., Kaushal S., Ojha U. (2020). Catalyst-Free
Partially Bio-Based
Polyester Vitrimers. ACS Appl. Polym. Mater..

[28] Cherif L. Y., Naar N., Mekki A., Ouadah A., Hamada R. A., Trache D. (2025). Unlocking the Potential of Polylactic Acid: Exploring
Its Thermal and Mechanical Properties for Vitrimerization via Double
Reactive Extrusion and Transesterification. J. Appl. Polym. Sci..

[29] Isogai T., Hayashi M. (2022). Critical Effects of Branch Numbers at the Cross-Link
Point on the Relaxation Behaviors of Transesterification Vitrimers. Macromolecules.

[30] Lemouzy S., Cuminet F., Berne D., Caillol S., Ladmiral V., Poli R., Leclerc E. (2022). Understanding
the Reshaping of Fluorinated
Polyester Vitrimers by Kinetic and DFT Studies of the Transesterification
Reaction. Chem. - Eur. J..

[31] Self J. L., Dolinski N. D., Zayas M. S., Read
De Alaniz J., Bates C. M. (2018). Brønsted-Acid-Catalyzed Exchange
in Polyester
Dynamic Covalent Networks. ACS Macro Lett..

[32] Dey I., Samanta K., Debnath T., Kundu D., Ghosh S., Bose S. (2025). Recycling
Reinvented: Converting PET Bottle Waste into 3D Printable
PCR Polypropylene Vitrimers for a Sustainable Future. Chem. Eng. J..

[33] Hayashi M., Inaba T. (2021). Achievement of a Highly Rapid Bond Exchange for Self-Catalyzed Polyester
Vitrimers by Incorporating Tertiary Amino Groups on the Network Strands. ACS Appl. Polym. Mater..

[34] Moazzen K., Rossegger E., Alabiso W., Shaukat U., Schlögl S. (2021). Role of Organic
Phosphates and Phosphonates in Catalyzing Dynamic Exchange Reactions
in Thiol-Click Vitrimers. Macromol. Chem. Phys..

[35] Danielson M. K., Gainaru C., Demchuk Z., Pan C., Choi J., Zhang H. H., Foster J. C., Saito T., Rahman M. A. (2025). Closed-Loop
Recyclable Vitrimer Plastics from PET Waste: A Design for Circularity. ChemSuschem.

[36] Sabeeh S., Algburi S., Alkhawlani Q., Jabbar F. I., El-Meligy M., Mahmoud H. A. (2025). Upcycled PET Vitrimer Filaments via Reactive Extrusion
for High Strength Heat Resistant and Repairable FDM Parts. Polym. Test..

[37] Tratnik N., Tanguy N. R., Davidson D. J., Yan N. (2025). Starch-Based Reversible
Adhesive: Effect of off-Stoichiometric Ratios on Transesterification
Vitrimer Properties. Carbohydr. Polym..

[38] Otera J. (1993). Transesterification. Chem. Rev..

[39] Cai W., Huang Y., Li J., Yang G., Wang F., Si G., Tan C. (2023). A Multifunctional
Biomass Zinc Catalyst for Epoxy-Based
Vitrimers and Composites. Eur. Polym. J..

[40] da
Cunha R. B., Brito L. B. Q., Agrawal P., Brito G. D. F., Mélo T. J. A. D. (2024). Recyclable Catalyst-Free Elastomeric
Vitrimers: Dynamic Covalent Bonds in Ethylene-Glycidyl Methacrylate/Zinc
Ionomer of Ethylene/Methacrylic Acid Blends. Macromolecules.

[41] Wang S., Gao S., Zhang X., Li X., Zhang Y. R. (2025). Reprocessable and
Scalable Ethylene Acrylic Acid Copolymer (EAA) Derived Vitrimer Based
on Dual Cross-Linked Networks. J. Polym. Environ..

[42] Demongeot A., Mougnier S. J., Okada S., Soulié-Ziakovic C., Tournilhac F. (2016). Coordination and Catalysis of Zn2+ in Epoxy-Based Vitrimers. Polym. Chem..

[43] Chen J., Wang S., Huan J., Li Z., Yang X., Li X., Tu Y. (2024). Effect of Coordination
Sacrificial Bond Strength on
Toughening Properties of Polyesters. Macromolecules.

[44] Hayashi M., Obara H., Shibata K., Sugimoto K., Takasu A. (2020). Glass Transition
Analysis of Model Metallosupramolecular Polyesters Bearing Pendant
Pyridine Ligands with a Controlled Ligand–Ligand Distance. Polym. J..

[45] Zhao Y., Mao H., Zhang T., Guo Z., Bai D., Bai H., Zhang Q., Xiu H., Fu Q. M. R. (2022). Heat-Resistant,
and Reprocessable Polyethylene Vitrimers Cross-Linked by β-Hydroxy
Ester Bonds via Reactive Processing. Ind. Eng.
Chem. Res..

[46] Zhao Z. H., Li C. H., Zuo J. L. (2023). Dynamic Polymeric
Materials Based
on Reversible B–O Bonds with Dative Boron–Nitrogen Coordination. SmartMater.

[47] Gosecki M., Gosecka M. (2022). Boronic Acid Esters
and Anhydrates as Dynamic Cross-Links
in Vitrimers. Polymer.

[48] Fang H., Zhao Y., Xie X., Zhang F., Xie X., Zhu J., Ren S., Ding Y. (2024). Enhancing Mechanical Performance
of Boronic Ester Based Vitrimers via Intermolecular Boron–Nitrogen
Coordination. Polymer.

[49] Ren S., Li Z., Zhou W., Zhu J., Zhao Y., Liu C., Fang H., Ding Y. S. R. (2023). Reprocessing and Self-Healing of
Castor Oil-Based Polyurethane Vitrimers Enabled by Supramolecular
Nitrogen-Coordinated Dynamic Covalent Boronic Ester. Ind. Crops Prod..

[50] Zych A., Tellers J., Bertolacci L., Ceseracciu L., Marini L., Mancini G., Athanassiou A. (2021). Self-Healing
Boronic Ester Vitrimers from Epoxidized Soybean Oil Acrylate. ACS Appl. Polym. Mater..

[51] Wu S. S., Li Y. D., Hu Z., Zeng J. B. (2023). Fast Upcycling of
Poly­(Ethylene Terephthalate) into Catalyst-Free Vitrimers. ACS Sustainable Chem. Eng..

[52] Zhao Y., Bai X., Zhang Y., Wang Y., Li Y., Yang S. (2025). Bio-Based
Recyclable Epoxy Vitrimers Prepared by The Combined Action of Cyclic
Boroxine and Acylhydrazone Bonds. J. Polym.
Environ..

[53] Zhang W., Cui X., Zhang H., Yang Y., Tang P. L. V. (2023). Nonlinear Rheology
and Applications of Polyethylene Terephthalate Vitrimers. J. Polym. Sci..

[54] Sarda P., Hanan J. C., Lawrence J. G., Allahkarami M. (2022). Sustainability
Performance of Polyethylene Terephthalate, Clarifying Challenges and
Opportunities. J. Polym. Sci..

[55] Li P., Lan B., Zhang X., Lei S., Yang Q., Gong P., Park C. B., Li G. (2022). Facile in
Situ Construction of a
Covalent Adaptable Network Polyester Vitrimer with Advanced Performance
in Repairability, Foamability and Recyclability. Green Chem..

[56] Zhang W., Zhang H., Yang Y., Tang P. (2024). Network Homogeneity
on Linear and Nonlinear Viscoelasticity of Polyethylene Terephthalate
Vitrimers. Polymer.

[57] Demongeot A., Groote R., Goossens H., Hoeks T., Tournilhac F., Leibler L. (2017). Cross-Linking of Poly­(Butylene Terephthalate)
by Reactive
Extrusion Using Zn­(II) Epoxy-Vitrimer Chemistry. Macromolecules.

[58] Panagiotopoulos C., Kapageridou E., Karamitrou M., Korres D. M., Charitidis C. A., Vouyiouka S. N. (2024). Recycling/Upcycling
of Physically Aged Poly­(Butylene
Succinate) into PBS Vitrimers through Zn­(II)-Catalyzed Melt Vitrimerization. Macromolecules.

[59] Zhou Y., Goossens J. G. P., Sijbesma R. P., Heuts J. P. A. (2017). Poly­(Butylene
Terephthalate)/Glycerol-Based Vitrimers via Solid-State Polymerization. Macromolecules.

[60] Thongkam M., Saowsupa S., Rungrojchaipon P. (2023). Vitrimer-like
of Poly (Glycerol Sebacate)
by Using Zinc Acetate, Zinc Oxide and Amberlyst as Catalysts. Curr. Res. Green Sustainable Chem..

[61] Soriano
Cuadrado B., Peñas Sanjuan A., Rodríguez
López J., Delgado Blanca I., Grande M. J., Lucas R., Galvez A., Pulido R. P. (2022). Effect of High-Pressure Treatments
on the Properties of Food Packaging Materials with or without Antimicrobials. Polymer.

[62] Xiao Y., Watson M. (2019). Guidance on Conducting a Systematic Literature Review. J. Plan. Educ. Res..

[63] Tranfield D., Denyer D., Smart P. (2003). Towards a Methodology
for Developing
Evidence-Informed Management Knowledge by Means of Systematic Review. Br. J. Manag..

[64] Chakma P., Konkolewicz D. (2019). Dynamic Covalent Bonds in Polymeric Materials. Angew. Chem..

[65] Krishnakumar B., Sanka R. V. S. P., Binder W. H., Parthasarthy V., Rana S., Karak N. (2020). Vitrimers: Associative Dynamic Covalent
Adaptive Networks in Thermoset Polymers. Chem.
Eng. J..

[66] Hayashi M., Uchiyama S., Kato M. (2023). Clarifying
the Role of Epoxy Molecules
and Base Catalysts for Vitrimer Transformation from Linear Polyesters. ACS Appl. Polym. Mater..

[67] Denissen W., Winne J. M., Du Prez F. E. (2016). Vitrimers:
Permanent Organic Networks
with Glass-like Fluidity. Chem. Sci..

[68] Awaja F., Pavel D. (2005). Recycling of PET. Eur. Polym. J..

[69] Maeda T., Otsuka H., Takahara A. (2009). Dynamic Covalent
Polymers: Reorganizable
Polymers with Dynamic Covalent Bonds. Prog.
Polym. Sci..

[70] Fortman D. J., Brutman J. P., De Hoe G. X., Snyder R. L., Dichtel W. R., Hillmyer M. A. (2018). Approaches to Sustainable
and Continually Recyclable
Cross-Linked Polymers. ACS Sustainable Chem.
Eng..

[71] Röttger M., Domenech T., Van Der Weegen R., Breuillac A., Nicolaÿ R., Leibler L. (2017). High-Performance Vitrimers
from Commodity
Thermoplastics through Dioxaborolane Metathesis. Science.

[72] Breuillac A., Caffy F., Vialon T., Nicolaÿ R. (2020). Functionalization
of Polyisoprene and Polystyrene via Reactive Processing Using Azidoformate
Grafting Agents, and Its Application to the Synthesis of Dioxaborolane-Based
Polyisoprene Vitrimers. Polym. Chem..

[73] He M., Lehn J. M. (2021). Metal Cation-Driven
Dynamic Covalent Formation of Imine
and Hydrazone Ligands Displaying Synergistic Co-Catalysis and Auxiliary
Amine Effects. Chem. – A Eur. J..

[74] Anagwu F. I., Thakur V. K., Skordos A. A. (2023). High-Performance
Vitrimeric Benzoxazines
for Sustainable Advanced Materials: Design, Synthesis, and Applications. Macromol. Mater. Eng..

[75] Berne D., Tanguy G., Caillol S., Poli R., Ladmiral V., Leclerc E. (2023). Transamidation Vitrimers Enabled by Neighbouring Fluorine
Atom Activation. Polym. Chem..

[76] Liang K., Zhang G., Zhao J., Shi L., Cheng J., Zhang J. M. (2021). Recyclable, and Robust Poly­(Amide–Imine)
Vitrimers
Prepared through a Green Polymerization Process. ACS Sustainable Chem. Eng..

[77] Kuhzadmohammadi A., Zhang N. (2025). Atomistic Modeling
of Bond Exchange Reaction and Self-Healing Mechanisms
in Epoxy Vitrimers. Eur. Polym. J..

[78] Liu H., Liu C., Liu Y., Jiang Y., Li X., Bai Y. (2024). Rapid Repair
and Degradation: A Study of High-Performance Recyclable Vitrimer Epoxy
Resin Based on Disulfide Bonds. Polym. Test.

[79] Millan A., Wodrinski A., Guerre M. (2025). Disulfide-Initiated Addition–Fragmentation
Chain Transfer in Allyl Sulfide-Based Vitrimers. Angew. Chem..

[80] Orrillo A. G., Furlan R. L. E. (2022). Sulfur in Dynamic
Covalent Chemistry. Angew. Chem., Int. Ed..

[81] Zhao J. Z., Yue T. J., Ren B. H., Lu X. B., Ren W. M. (2024). Closed-Loop
Recycling of Sulfur-Rich Polymers with Tunable Properties Spanning
Thermoplastics, Elastomers, and Vitrimers. Nat.
Commun..

[82] Tratnik N., Tanguy N. R., Yan N. R. (2023). Self-Strengthening Starch-Based Epoxy
Vitrimer Facilitated by Exchangeable Disulfide Bonds. Chem. Eng. J..

[83] Wang J., You W., Chen L., Xiao D., Xiao X., Shan T., Liu Y., Liu M., Li G., Yu W., Huang F. (2024). Adaptive and
Robust Vitrimers Fabricated by Synergy of Traditional and Supramolecular
Polymers. Angew. Chem., Int. Ed..

[84] Zhang S., Jiang C., Wang C., Li J., Ma Y., Zhu Y., Deng H., Zhao Y., Zhang G. (2024). Reprocessable and Self-Healable
Boronic-Ester-Based SBS Vitrimers with Improved Thermomechanical Property
and Adhesive Performance. Ind. Eng. Chem. Res..

[85] Biswas S., Das P., Chakraborty S., Nayak J., Haridas Cp C. A., Katheria A., Mondal T., Ray S. K., Das N. C., Das N. C. (2025). Multifunctional
Conductive Elastomeric Composites for
Broadband Electromagnetic Radiation Shielding and Biomechanical Energy
Harvesting. ACS Appl. Polym. Mater..

[86] Das P., Katheria A., Jana A., Das M., Roy B., Nayak J., Nath K., Ghosh S. K., De A., Das N. C.-S.-S. (2023). Self-Healing 2D MXene-Based Composites for Thermal
Management and Electromagnetic Shielding Applications. ACS Appl. Eng. Mater..

[87] Chen Z., Jin C., Li Z., Li X., Yang L., Wang T. (2023). Tough Epoxy
Vitrimer with Hybrid Networks and Its Applications in Reprocessable
Composites. Adv. Eng. Mater..

[88] Song Z., Wang Z., Cai S. (2021). Mechanics
of Vitrimer with Hybrid
Networks. Mech. Mater..

[89] Zhang X., Wang S., Jiang Z., Li Y., Jing X. (2020). Boronic Ester
Based Vitrimers with Enhanced Stability via Internal Boron–Nitrogen
Coordination. J. Am. Chem. Soc..

[90] Jiang C., Yan H., Wang C., Zhang S., Zhu Y., Deng H., Zhao Y., Zhang G. (2025). Boronic-Ester-Based SBS Vitrimer
with a Synergistic Zn2+-Coordinated Network Toward Mechanically Robust
and Malleable Elastomers. Ind. Eng. Chem. Res..

[91] Choe Y. A., Pak R. B., Kim S. I., Ju K. S. (2023). Preparation and
Characterization of Water-Reducible Polyester Resin Based on Waste
PET for Insulation Varnish. RSC Adv..

[92] Di
Lorenzo M. L. (2024). Crystallization of Poly­(Ethylene Terephthalate): A
Review. Polym.

[93] Zhu P., Dezhu D. (1999). Study on the Double
Cold Crystallization Peaks of Poly­(Ethylene Terephthalate)
(PET): 2. Samples Isothermally Crystallized at High Temperature. Eur. Polym. J..

[94] Wang Z. G., Hsiao B. S., Sauer B. B., Kampert W. G. (1999). The Nature
of Secondary
Crystallization in Poly­(Ethylene Terephthalate). Polymer.

[95] Valdez S., Qiang Z. (2025). Understanding Non-Isothermal Crystallization
Behaviors of Polyethylene-Derived
Vitrimers. Macromolecules.

[96] Ricarte R. G., Tournilhac F., Leibler L. (2019). Phase Separation and Self-Assembly
in Vitrimers: Hierarchical Morphology of Molten and Semicrystalline
Polyethylene/Dioxaborolane Maleimide Systems. Macromolecules.

[97] Soman B., Go Y. K., Shen C., Leal C., Evans C. M. (2022). Impact
of Dynamic Covalent Chemistry and Precise Linker Length on Crystallization
Kinetics and Morphology in Ethylene Vitrimers. Soft Matter..

[98] Kuenstler A. S., Bowman C. N. (2023). Catalytic Control of Crystallization in Dynamic Networks. ACS Macro Lett..

[99] Soman, B. Investigating the effect of dynamic covalent bonds on the crystallization, Rheology And Molecular Relaxation In Ethylene Vitrimers; University of Illinois Urbana-Champaign, 2022.

[100] Chen C. W., Mao H. I., Lee H. K., Chou J. Y., Rwei S. P. (2024). Upcycling Recycled Epoxy-Based Vitrimer for Enhancing
Toughness of Poly­(Ethylene Terephthalate). J.
Polym. Environ..

[101] Chen Z., Hay J. N., Jenkins M. J. (2013). The Thermal Analysis
of Poly­(Ethylene Terephthalate) by FTIR Spectroscopy. Thermochim. Acta..

[102] Allen N. S., Edge M., Mohammadian M., Jones K. (1991). Hydrolytic Degradation of Poly­(Ethylene Terephthalate): Importance
of Chain Scission versus Crystallinity. Eur.
Polym. J..

[103] Lv G., Li X., Jensen E., Soman B., Tsao Y. H., Evans C. M., Cahill D. G. (2023). Dynamic Covalent
Bonds in Vitrimers
Enable 1.0 W/(m K) Intrinsic Thermal Conductivity. Macromolecules.

[104] Nettles J. A., Long T. E., Jin K. (2026). Reactive Melt Extrusion
for Polymer Deconstruction and Upcycling. JACS
Au.

[105] Cassagnau P., Bounor-Legaré V., Vergnes B. (2019). Experimental
and Modelling Aspects of the Reactive Extrusion Process. Mech. Ind.

[106] Poutrel Q. A., Kmo R., Cohadon A., Boisse J., Rouzière S., André S., Hoppe S., Farge L. (2024). Effect of
Processing Conditions on the Properties of Vitrimerized Polybutylene
Terephthalate Prepared by Reactive Extrusion. Polym. Degrad. Stab.

[107] Rashwan O., Koroneos Z., Townsend T. G., Caputo M. P., Bylone R. J., Wodrig B., Cantor K. (2023). Extrusion and Characterization
of Recycled Polyethylene Terephthalate (RPET) Filaments Compounded
with Chain Extender and Impact Modifiers for Material-Extrusion Additive
Manufacturing. Sci. Rep.

[108] Daver F., Gupta R., Kosior E. (2008). Rheological
Characterisation
of Recycled Poly­(Ethylene Terephthalate) Modified by Reactive Extrusion. J. Mater. Process. Technol.

[109] Jaime-Azuara A., Longo E., Boselli E., Baratieri M., Pedersen T. H. (2024). Exploratory DSC Investigation on
the Solvolytic Depolymerization
of PET in Varied Solvent Systems and in the Presence of Model Additives
and Contaminants. Polym. Degrad. Stab.

[110] Farge L., Hoppe S., Daujat V., Tournilhac F., Andre S. (2021). Solid Rheological Properties of PBT-Based
Vitrimers. Macromolecules.

[111] Xanthos M., Wan C., Dhavalikar R., Karayannidis G. P., Bikiaris D. N. (2004). Identification of Rheological and
Structural Characteristics of Foamable Poly­(Ethylene Terephthalate)
by Reactive Extrusion. Polym. Int..

[112] Awaja F., Daver F., Kosior E. (2004). Recycled Poly­(Ethylene
Terephthalate) Chain Extension by a Reactive Extrusion Process. Polym. Eng. Sci..

[113] Awaja F., Daver F., Kosior E., Cser F. (2004). The Effect
of Chain Extension on the Thermal Behaviour and Crystallinity of Reactive
Extruded Recycled PET. J. Therm. Anal. Calorim..

[114] Incarnato L., Scarfato P., Di Maio L., Acierno D. (2000). Structure
and Rheology of Recycled PET Modified by Reactive Extrusion. Polymer.

[115] Ragaert K., Delva L., Van Geem K. (2017). Mechanical
and Chemical
Recycling of Solid Plastic Waste. Waste Manage..

